# Implementing an isoniazid preventive therapy program for people living with HIV in Thailand

**DOI:** 10.1371/journal.pone.0184986

**Published:** 2017-09-26

**Authors:** Junya Danyuttapolchai, Somyot Kittimunkong, Sriprapa Nateniyom, Sutthapa Painujit, Virat Klinbuayaem, Nuanpun Maipanich, Yongyut Maokamnerd, Eric Pevzner, Sara Whitehead, Apiratee Kanphukiew, Patama Monkongdee, Michael Martin

**Affiliations:** 1 Thailand Ministry of Public Health–U.S. Centers for Disease Control and Prevention Collaboration, Nonthaburi province, Thailand; 2 Bureau of Vector-Borne Diseases, Department of Disease Control, Thailand Ministry of Public Health, Nonthaburi province, Thailand; 3 Bureau of Tuberculosis, Department of Disease Control, Thailand Ministry of Public Health, Bangkok, Thailand; 4 Nakhon Si Thammarat Hospital, Nakhon Si Thammarat Province, Thailand; 5 Sanpatong Hospital, Chiang Mai Province, Thailand; 6 Vachira Phuket Hospital, Phuket Province, Thailand; 7 Somdejprajaotaksin Maharaj Hospital, Tak Province, Thailand; 8 U. S. Centers for Disease Control and Prevention, Atlanta, Georgia, United States of America; Agencia de Salut Publica de Barcelona, SPAIN

## Abstract

Treatment of people living with HIV (PLHIV) with latent tuberculosis (TB) infection using isoniazid preventive therapy (IPT) can reduce the risk of TB disease, however, the scale-up of IPT among PLHIV in Thailand and worldwide has been slow. To hasten the implementation of IPT in Thailand, we developed IPT implementation training curricula and tools for health care providers and implemented IPT services in seven large government hospitals. Of the 659 PLHIV enrolled, 272 (41.3%) reported symptoms of TB and 39 (14.3% of those with TB symptoms) were diagnosed with TB. A total of 346 (52.4%) participants were eligible for IPT; 318 (91.9%) of these participants opted to have a tuberculin skin test (TST) and 52 (16.3% of those who had a TST) had a positive TST result. Among the 52 participants with a positive TST, 46 (88.5%) initiated and 39 (75.0%) completed 9 months of IPT: physicians instructed three participants to stop IPT, two participants were lost to follow-up, one chose to stop therapy, and one developed TB. IPT can be implemented among PLHIV in Thailand and could reduce the burden of TB in the country.

## Introduction

Thailand, an upper-middle income country in Southeast Asia with a population of 68 million, had an estimated tuberculosis (TB) incidence of 172 cases per 100,000 people in 2015 making it one of 30 countries in the world classified by the World Health Organization (WHO) as having the highest burden of TB [1]. Among people diagnosed with TB in Thailand in 2015, an estimated 13% were also infected with HIV [1]. TB is the leading cause of death among people living with HIV (PLHIV), and HIV infection increases the risk of rapidly progressing from TB infection to TB disease [2]. Antiretroviral therapy (ART) reduces, but does not eliminate the risk of TB disease among PLHIV [3–5]. Treatment of people with latent TB infection using isoniazid preventive therapy (IPT) can reduce the risk of latent TB infection progressing to TB disease by 60% to 90% [6]. IPT offers protection from TB disease above and beyond the protection afforded by ART [7]. Therefore, the WHO recommends IPT for PLHIV as part of a TB prevention package that includes infection control practices for TB and intensified case-finding of TB (i.e., the Three I’s) [8–9].

Despite evidence that IPT reduces TB incidence in PLHIV and WHO recommendations to incorporate IPT as part of comprehensive TB and HIV activities, scale-up of IPT among PLHIV worldwide has been slow [10]. Implementation of IPT programs is challenging for several reasons: careful coordination of activities is required among national and local TB and HIV programs and clinics, PLHIV eligible for IPT are often identified in HIV clinics and may need referral to TB clinics for clinical assessment and provision of IPT, and supply chain management and follow-up care and monitoring issues can be complicated if they are not standardized.

In Thailand, IPT is recommended for PLHIV who have latent TB infection based on a positive tuberculin skin test (TST) [11]. WHO recommendations do not require a TST to initiate IPT among PLHIV [8]; however, because studies have shown significant reductions in incident TB and mortality among PLHIV with positive TST results and not among PLHIV with negative TST results, the Thai government recommends TST [5,12]. In Thailand and many parts of the world where TB is common, Bacillus Calmette-Guerin (BCG) vaccine is used to protect infants and young children from serious life-threatening disease [13]. BCG vaccine can cause a positive TST response, but this response generally wanes over time and, in Thailand, a TST response showing induration of ≥5 millimeters (mm) in PLHIV is defined as a positive TST response [11–14].

In order to hasten the implementation of IPT among PLHIV nationwide, Thailand’s Ministry of Public Health (MOPH) organized an IPT Guideline Committee with representatives from the MOPH Bureau of TB, the MOPH Chest Institute and Infectious Diseases Hospital, the Thai Red Cross, University Hospitals, and the U.S. Centers for Disease Control and Prevention (CDC). The IPT Guideline Committee met in 2009 to review and align existing IPT recommendations for PLHIV, develop IPT implementation training tools for health care providers, and organize sustainable systems to deliver and store TST reagent and isoniazid.

An IPT protocol was developed to guide implementation of IPT in selected hospitals in Vietnam and Thailand. We identified seven large government hospitals with existing TB and HIV services in Thailand to implement IPT services for PLHIV using the curricula and tools developed by the Committee. Here, we report the results of the IPT program in Thailand two years after implementation.

## Methods

### Development of curricula and tools

The IPT Guideline Committee reviewed existing national TB recommendations including the 2009 MOPH National HIV/TB Guidelines [15], the 2008 National Tuberculosis Control Programme Guidelines [16], and the 2003 Implementation Manual for Treatment of PLHIV with latent TB infection in Northern Thailand [17] to develop training tools. The draft IPT guidelines recommended 9 months IPT for PLHIV with latent TB infection and the staff training tools included information on the evidence supporting the use of IPT among PLHIV, how to screen PLHIV for TB disease and latent TB infection, how to record information gathered during the screening process, how to monitor PLHIV receiving IPT, and hands-on training about TST administration.

The Thailand Vietnam IPT protocol recommended that participants with TB symptoms who completed an evaluation for TB disease and were found not to have TB disease, be evaluated for IPT; however, in Thailand, the IPT Guideline Committee recommended that only people with a negative TB symptom screen be evaluated for IPT. Consistent with the guideline committee recommendation, site staff at the seven hospitals in Thailand were instructed to only evaluate participants with a negative TB symptom screen for IPT.

### Study population

Seven hospitals were selected because they had well established HIV and TB treatment programs. In these hospitals, TB services (i.e., screening, diagnosis, and treatment) were provided in the HIV clinic. The hospitals in Chiang Rai (756 beds), Nakhon Si Thammarat (1000 beds), Phitsanulok (904 beds), and Phuket (534 beds) are urban, tertiary care, regional hospitals; Srisaket Hospital (506 beds) is an urban, tertiary care provincial hospital; Tak Hospital (310 beds) is an urban, secondary care provincial hospital; and Chiang Mai Hospital (456 beds) is a rural, secondary care district hospital. At the outpatient HIV clinics in these seven hospitals, staff offered PLHIV ≥18 years newly registered for HIV care or previously registered and being evaluated for ART initiation an assessment for TB disease and latent TB infection.

### Ethical considerations

Patients interested in participating in the study signed an informed consent form. The study protocol was approved by the Ethical Review Committees of Nakhon Si Thammarat Hospital, Phitsanulok Hospital, Chaing Rai Hospital, the Thailand MOPH, and the Institutional Review Board of CDC and the study was conducted according to the principles expressed in the Declaration of Helsinki.

### Site preparation

In 2010, we trained approximately 25 staff (i.e., HIV and TB clinic staff, laboratorians, pharmacists, and social medicine staff) from the seven hospitals about latent TB, TB disease, and how to implement IPT among PLHIV using the tools developed by the IPT Guideline Committee (S1 File. Workshop on IPT and TST). Supply chains for TST reagents and isoniazid were developed or strengthened at each hospital.

### Participant evaluation

From July 2010 through August 2011, staff screened patients for TB disease using an evidence-based TB symptom screening questionnaire [18]. The TB screening consisted of three questions: in the previous four weeks have you had 1) a cough or 2) a fever of any duration, or 3) drenching night sweats lasting three or more weeks. Participants with newly diagnosed HIV infection; or with fever, cough, or night sweats; or with clinical findings suggestive of TB were referred for a chest x-ray. Participants with one or more TB symptoms or an abnormal chest x-ray were sent for further evaluation including microbiologic examination of sputum and TB treatment if diagnosed with TB disease. Participants with a negative TB symptom screen, a normal chest x-ray (i.e., if a chest x-ray was done), and no history of TB disease or IPT use, liver disease, or an opportunistic infection were offered a TST. Participants with ≥5 mm of induration at the site of the TST 48 to 72 hours after placement were offered isoniazid 300 mg daily for 9 months. PLHIV receiving IPT were monitored at one month, and if there was no evidence of isoniazid toxicity, adverse drug reactions, or TB disease, every three months thereafter. Liver enzymes were examined at baseline and six months. Participants were followed for one year.

### Data collection and analysis

Staff used an interviewer-administered questionnaire with participants to collect demographic and risk behavior data, information about signs or symptoms of TB, liver disease, and opportunistic infection, and a history of TB or IPT at baseline and at one year. Staff abstracted laboratory and clinical data from participants’ medical records. Health care providers completed a questionnaire after the initial evaluation and usually within one month describing the findings of the evaluation and classifying participants as having TB disease or not having TB disease. We used this classification to define TB disease cases in the analysis. Approximately one year after the initial evaluation, research staff contacted health care providers for information about the people evaluated; several additional cases of TB disease were reported. We did not include these cases in the analysis. We used the chi-square and Fisher’s exact tests to test for differences in proportions. We used generalized estimating equations logistic regression to account for possible site level clustering and to explore participant characteristics associated with TB disease and a positive TST result. We included all variables in multivariable analyses. We used SPSS for Windows 18.0 (SPSS Inc. Chicago, Illinois, USA) and SAS version 9.3 (SAS Institute, North Carolina, USA) for analysis.

## Results

### Participants

**Fig 1:**
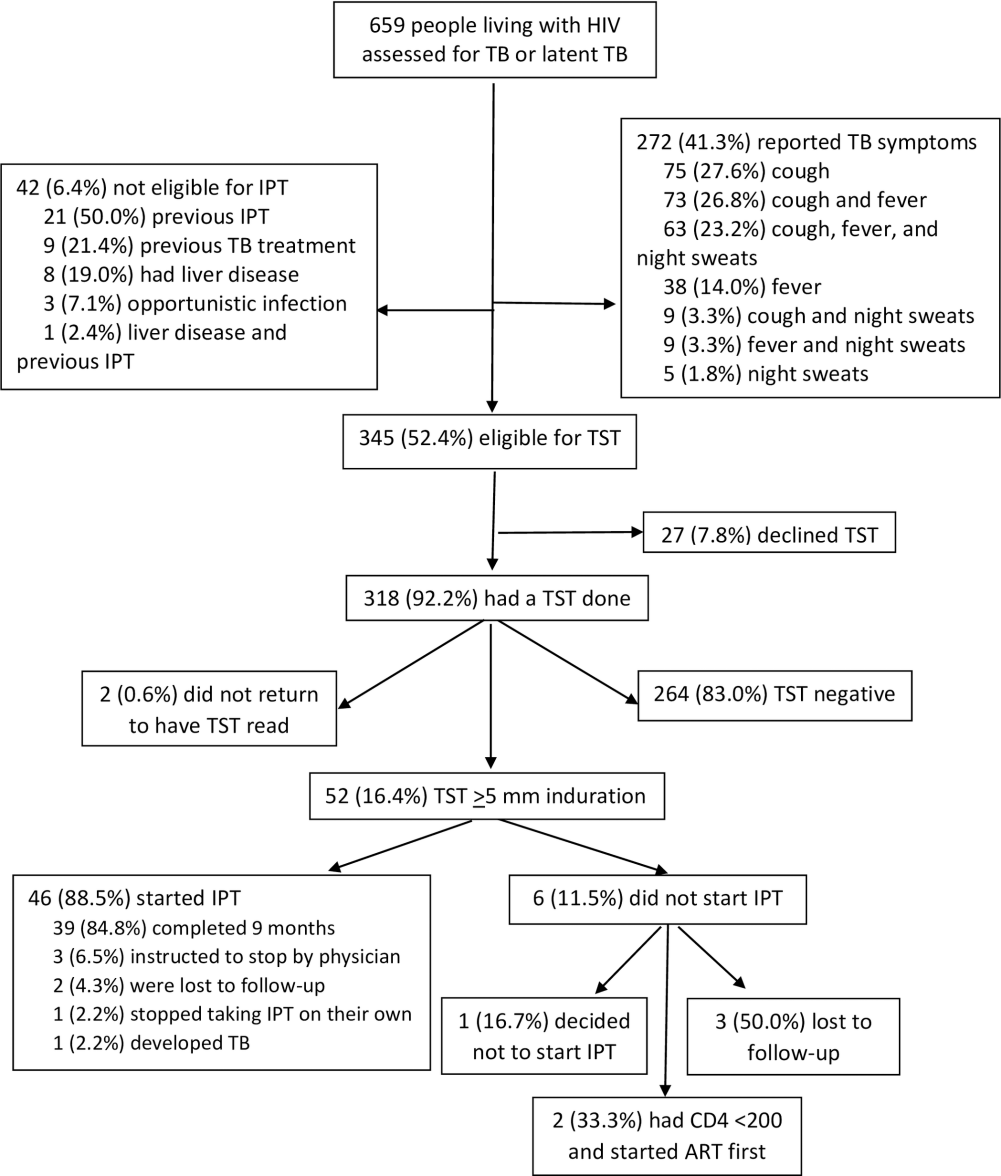
Participant flow.

From July 2010 through August 2011, 659 PLHIV ≥18 years not yet receiving ART enrolled in the study (Fig 1). A total of 272 (41.3%) participants reported symptoms associated with TB (i.e., in the previous four weeks had a cough or a fever of any duration, or drenching night sweats lasting three or more weeks). Men (47.0%) were more likely to have TB symptoms than women (35.6%) (p = 0.003), PLHIV who weighed <60 kilograms (46.0%) were more likely to have TB symptoms than those who weighed ≥60 kilograms (32.3%) (p = 0.0007), and PLHIV with CD4 counts <200 cells/mm^3^ (62.0%) were more likely to have TB symptoms than those who had CD4 counts ≥200 cells/mm^3^ (32.8%) (p<0.0001) (Table 1).

**Table 1:** Baseline characteristics of people living with HIV assessed for symptoms of tuberculosis* at seven hospitals in Thailand, 2010–2011.

Characteristics	All participants (N = 659) n (%)	Had TB symptoms* (n = 272) n (%)	No TB symptoms* (n = 387) n (%)	P value^†^
Age				
18–34 years	273 (41.4)	120 (44.0)	153 (56.0)	
≥35 years	386 (58.6)	152 (39.4)	234 (60.6)	0.24
Median age, in years (interquartile range [IQR])	36 (31–43)	36 (31–43)	36 (31–42)	
Sex				
Male	330 (50.1)	155 (47.0)	175 (53.0)	
Female	329 (59.9)	117 (35.6)	212 (64.4)	0.003
Body weight (kg)				
<60	433 (65.7)	199 (46.0)	234 (54.0)	
≥60	226 (34.3)	73 (32.3)	153 (67.7)	0.0007
CD4 count (cells/mm^3^)				
≥200	467 (70.9)	153 (32.8)	314 (67.2)	
<200	192 (29.1)	119 (62.0)	73 (38.0)	<0.0001
Median CD4 count, cells/mm^3^ (IQR)	312 (157–474)	231 (66–412)	358 (245–498)	
Currently smokes cigarettes				
No	504 (76.5)	200 (39.7)	304 (60.3)	
Yes	155 (23.5)	72 (46.4)	83 (53.6)	0.13
Drinks alcohol^‡^				
No	571 (86.6)	238 (41.7)	333 (58.3)	
Yes	88 (13.4)	34 (38.6)	54 (61.4)	0.59
Province				
Chiang Mai	119 (18.1)	71 (59.7)	48 (40.3)	
Chiang Rai	229 (34.8)	70 (30.6)	159 (69.4)	
Nakhon Si Thammarat	80 (12.1)	41 (51.2)	39 (48.8)	
Phitsanulok	78 (11.8)	36 (46.2)	42 (53.8)	
Phuket	73 (11.1)	14 (19.2)	59 (80.8)	
Srisaket	36 (5.5)	36 (100.0)	0 (0.0)	
Tak	44 (6.7)	4 (9.1)	40 (90.9)	<0.0001

HIV, human immunodeficiency virus; TB, tuberculosis

^*^Had at least one of three TB symptoms in the previous four weeks: cough, fever, and/or drenching night sweats lasting for three or more weeks.

^**†**^Chi-square test assessing characteristics associated with TB symptoms.

^‡^Participant drinks alcohol every day or the participant consumes five or more alcohol containing drinks at one time at least once per week.

### Participants with TB symptoms

Among the 272 participants with TB symptoms, 39 (14.3%) were diagnosed with TB disease: 15 (38.5%) had *Mycobacterium tuberculosis* (MTB) identified in sputum cultures, 12 (30.8%) had abnormal chest x-ray results without acid fast bacilli identified in sputum smears and were diagnosed with TB, seven (17.9%) had abnormal chest x-ray results, acid fast bacilli identified in sputum smears, and negative MTB cultures, three (7.7%) had MTB isolated from a lymph node, one (2.6%) had MTB isolated from pleura, and one (2.6%) was diagnosed with TB meningitis. In multivariable analysis, participants diagnosed with TB disease were more likely to be ≥35 years old (Odds Ratio (OR) 2.4, 95% confidence interval (CI) 1.0–5.7) and have a CD4 count <200 cells/mm^3^ (OR 2.5, 95% CI 1.4–4.4) and less likely to be female (OR 0.4, 95% CI 0.1–1.0) (Table 2).

**Table 2:** Results of generalized estimating equations logistic regression analysis of characteristics of people living with HIV with tuberculosis symptoms* to control for site-level clustering and to determine predictors of TB disease diagnosis at seven hospitals in Thailand, 2010–2011.

Characteristics (n = 272)	Diagnosed with TB (n = 39) n (%)	Bivariable analysis		Multivariable analysis	
		Odds Ratio (95% CI)	P-value	Odds Ratio (95% CI)	P-value
Age					
19–34 years (n = 120)	10 (8.3)	1.0		1.0	
≥35 years (n = 152)	29 (19.1)	2.6 (1.1–5.9)	0.02	2.4 (1.0–5.7)	0.05
Sex					
Male (n = 155)	30 (19.4)	1.0		1.0	
Female (n = 117)	9 (7.7)	0.3 (0.2–0.8)	0.01	0.4 (0.1–1.0)	0.05
Body weight (kilograms)					
<60 (n = 199)	32 (16.1)	1.0		1.0	
≥60 (n = 73)	7 (9.6)	0.6 (0.3–1.2)	0.12	0.6 (0.3–1.2)	0.15
CD4 count (cells/mm^3^)					
≥200 (n = 153)	13 (8.5)	1.0		1.0	
<200 (n = 119)	26 (21.9)	3.0 (2.0–4.6)	<0.0001	2.5 (1.4–4.4)	0.002
Currently smoke cigarettes					
No (n = 200)	27 (13.5)	1.0		1.0	
Yes (n = 72)	12 (16.7)	1.3 (0.6–2.5)	0.48	0.9 (0.4–2.5)	0.91
Drinks alcohol^**†**^					
No (n = 238)	34 (14.3)	1.0		1.0	
Yes (n = 34)	5 (14.7)	1.0 (0.7–1.6)	0.87	1.0 (0.7–1.3)	0.74

HIV, human immunodeficiency virus; TB, tuberculosis

*Had at least one of three TB symptoms in the previous four weeks: cough, fever, and/or drenching night sweats lasting for three or more weeks.

^**†**^ Participant drinks alcohol every day or the participant consumes five or more alcohol containing drinks at one time at least once per week.

One participant did not report fever, cough, or night sweats but was diagnosed with TB two months after the TB screening assessment (described below). Assuming this participant was infected with TB before the screening questions were asked, the sensitivity of the TB symptom screening questionnaire was 97.5%, the specificity was 62.5%, the positive predictive value was 14.3%, and the negative predictive value was 97.2%. Participants who had cough and fever or cough, fever and drenching night sweats accounted for 28 (71.8%) of participants diagnosed with TB (Table 3).

**Table 3:** Assessment of 272 people living with HIV who reported tuberculosis symptoms at baseline.

TB symptom or symptoms	Diagnosed with TB N = 39 n (%)	Not diagnosed with TB N = 233 n (%)	Total N = 272 n (%)
Cough only	6 (8.0)*	69 (92.0)*	75 (27.6) ^**†**^
Cough and fever	12 (16.4)	61 (83.6)	73 (26.8)
Cough, fever, and drenching night sweats	16 (25.4)	47 (74.6)	63 (23.2)
Fever only	2 (5.3)	36 (94.7)	38 (14.0)
Fever and drenching night sweats	1 (11.1)	8 (88.9)	9 (3.3)
Drenching night sweats only	0 (0)	5 (100)	5 (1.8)
Drenching night sweats and cough	2 (22.2)	7 (77.8)	9 (3.3)

*Row percent.

^**†**^Column percent.

### Testing for latent TB infection

Among the 387 (58.7%) participants who did not have symptoms of TB disease, 42 (10.8%) were not eligible for IPT. The most common reasons for being ineligible for IPT were previous IPT (n = 21, 50.0%) and previous TB treatment (n = 9, 21.4%). A total of 345 (52.4%) participants were eligible for IPT and offered a TST and 318 (92.2%) of these participants opted to have a TST done (Fig 1).

The median age of the 318 participants who had a TST placed was 36 years (interquartile range 30–42 years) and 178 (56.0%) were women. Two (0.6%) participants did not return for staff to read the TST result and 52 (16.4%) had a positive TST (≥5 mm of induration). None of the 28 participants in Chiang Mai or the 39 participants in Phitsanulok had a positive TST; while 15 (42.9%) of 35 in Nakhon Si Thammarat, 10 (25.0%) of 40 in Tak, 11 (20.4%) of 54 in Phuket, and 16 (13.2%) of 121 participants in Chiang Rai, and had positive TST results. Participants with positive TST results did not differ significantly from those with negative TST results by age, sex, weight, or CD4 count (Table 4).

**Table 4:** Results of generalized estimating equations logistic regression analysis of baseline characteristics of 316 people living with HIV who had a tuberculin skin test (TST) to determine predictors of a positive TST result (≥5mm of induration).

Characteristics (n = 316)	TST-positive n = 52 n (%)	Bivariable analysis		Multivariable analysis	
		Odds Ratio (95% CI)	P-value	Odds Ratio (95% CI)	P-value
Age (years)					
19–34 years (n = 131)	25 (19.1)	1.0		1.0	
>34 years (n = 185)	27 (14.6)	0.7 (0.4–1.2)	0.22	0.8 (0.5–1.2)	0.28
Sex					
Male (n = 139)	25 (18.0)	1.0		1.0	
Female (n = 177)	27 (15.3)	0.8 (0.7–1.0)	0.07	0.7 (0.3–1.3)	0.25
Weight (kg)					
<60 (n = 188)	32 (17.0)	1.0		1.0	
≥60 (n = 128)	20 (15.6)	0.9 (0.6–1.3)	0.56	0.8 (0.5–1.3)	0.46
CD4 count (cells/mm^3^)					
≥200 (n = 264)	44 (16.7)	1.0		1.0	
<200 (n = 52)	8 (15.4)	0.9 (0.4–2.3)	0.84	0.9 (0.4–2.2)	0.82
Currently smokes cigarettes					
No (n = 248)	42 (16.9)	1.0		1.0	
Yes (n = 68)	10 (14.7)	0.8 (0.3–2.2)	0.73	0.8 (0.3–2.3)	0.67
Drinks alcohol*					
No (n = 274)	48 (17.5)	1.0		1.0	
Yes (n = 42)	4 (9.5)	0.5 (0.1–2.5)	0.39	0.5 (0.1–2.4)	0.37

CI, confidence interval; kg, kilograms.

^*^Participant drinks alcohol every day or the participant consumes five or more alcohol containing drinks at one time at least once per week.

### Isoniazid preventive therapy

Among the 52 participants with a positive TST, one decided not to start IPT, two had CD4 count results less than 200 cells/mm^3^ and their doctors decided to start ART first, and three participants were lost to follow-up before being offered IPT (Fig 1). A total of 46 (88.5%) participants initiated IPT and 39 (75.0%) completed nine months of IPT. Three (6.5%) participants were instructed to stop IPT by their physicians (two had elevated aminotransferase results and one had a skin rash), two (4.3%) were lost to follow-up, one (2.2%) stopped taking IPT on their own, and one (2.2%) developed TB disease. The participant who developed TB disease had a normal chest x-ray, a CD4 count of 333 cells/mm^3^, and a TST of 20 mm at baseline. The participant returned to the clinic after 2 months of IPT with a fever, had an abnormal chest x-ray and a negative sputum smear for TB, but was empirically diagnosed and started on treatment for TB disease. The participant completed TB treatment without complications and was alive and well at the one year follow-up visit.

### Adverse drug reactions

One participant developed a skin rash that was not described in detail in the medical record. The participant’s physician stopped all medicines including isoniazid and ART, the rash subsided, and ART was restarted. Two participants had elevated alanine aminotransferase results and their physicians stopped isoniazid. Both participants were alive and well, and had not restarted IPT at the one year follow-up visit.

## Discussion

Hospital staff assessed 659 PLHIV and found that 52.4% were eligible for a TST; 16.4% of the 318 who chose to have a TST tested positive for latent TB infection. Of those diagnosed with latent TB infection 75.0% completed 9 months of IPT. These findings demonstrate that with appropriate preparation, training, and support hospital staff in Thailand can successfully implement IPT among PLHIV. Among PLHIV who reported TB symptoms in the screening assessment (i.e., in the previous four weeks had a cough or a fever of any duration, or drenching night sweats lasting three or more weeks) 14.3% were diagnosed with TB disease. The sensitivity, specificity, and predictive values of the three questions TB screening algorithm among participants in this study were similar to results reported in the literature [18].

In 2014, only 13 of 41 high TB/HIV burden countries reported to WHO that IPT had been provided to PLHIV to prevent TB disease. Among the 13 countries reporting to WHO, IPT coverage ranged from 5% in Swaziland to 97% in Haiti [1]. Several studies have assessed IPT among PLHIV at sites in Thailand. In a study in northern Thailand in the mid-1990s among 412 PLHIV with positive TST results, 286 (69.4%) completed 9 months of IPT; female participants and participants who were married, widowed, or self-employed were more likely to be adherent to IPT [14]. A similar study at an AIDS research center in Bangkok during 2003–2008 among 799 participants with positive TST results, found that 551 (69.0%) completed 9 months of IPT and participants with baseline CD4 counts >200 cells/mm^3^ were more likely to complete 9 months of IPT [19]. Consistent with these studies, we found high uptake (88.5%) and completion (75.0%) of IPT among PLHIV and use of TST reduced the number of PLHIV targeted for IPT from 346 (52.4%) to 52 (7.9%). Despite evidence that IPT can reduce the burden of TB and WHO recommendations to provide IPT to PLHIV, health care worker concerns about excluding TB disease, creating isoniazid resistance, hepatitis, and IPT adherence have limited the scale-up IPT nationally in Thailand [20].

There were an estimated 455,000 PLHIV in Thailand in 2014 [21]. Based on our findings (i.e., 318 [48.3%] of the 659 PLHIV evaluated were eligible for and opted to have a TST placed and 52 [16.3%] of these participants had a positive TST result), approximately 36,000 PLHIV would have a positive TST result and be eligible for IPT. A study in New York City in 1985–1987 found that PLHIV with positive TST results developed TB disease at a rate of 7.9 per 100 person-years [2], suggesting that 2,800 cases of TB could potentially be prevented in Thailand each year if IPT were made available to PLHIV. Thailand is one of 30 countries in the world with the highest burden of TB and TB causes substantial morbidity and mortality in the country, particularly among PLHIV. Assessing PLHIV for TB disease and providing IPT to those who are eligible would reduce the incidence of TB among PLHIV, prevent new infections, and thereby reduce the burden of TB in Thailand.

Our report has several limitations. We implemented the IPT activity in hospitals that had well developed HIV and TB services; other facilities may not be able to provide the same level of care. In settings where TST is not available, IPT should be considered for all PLHIV as recommended by WHO [8]. We did not use new molecular diagnostic laboratory technologies. These automated diagnostic tools can decrease the time to diagnosis of TB and drug resistant TB, have improved sensitivity for diagnosing disease, and may provide healthcare workers with greater confidence that they have excluded TB disease and can safely initiate IPT among PLHIV [22]. Participants who did not report fever, cough, or night sweats and did not have clinical findings or chest x-ray results suggestive of TB were not systematically evaluated for TB with sputum microbiologic exam. Thus, some cases of TB disease may have been missed in this population.

Based on the recommendations of the National IPT Guideline Committee and the successful implementation of IPT in seven hospitals, the Thailand MOPH recommended that all PLHIV be assessed for and offered IPT for latent TB infection in the 2013 National Tuberculosis Control Guidelines [11] and included the treatment recommendations for PLHIV with latent TB infection in the Thailand National Guidelines on HIV/AIDS issued in 2017 [23]. The capacity of hospital staff to implement TB screening and IPT will need to be increased to make comprehensive TB services accessible to all PLHIV in Thailand.

## Supporting information

S1 Table Data. (XLSX)

S1 File Workshop on IPT and TST. S1 File contains a workshop agenda, sample of overview ICF-IPT project in Thailand, sample of IPT study, and sample of latent TB infection and TST. (PDF) **Figure A.** Workshop agenda. (PDF) **Figure B.** Sample of overview ICF-IPT project in Thailand. (PDF) **Figure C.** Sample of IPT study. (PDF) **Figure D.** Sample of latent TB infection and TST.
(ZIP)
