# Supplementary material for: Implementing an isoniazid preventive therapy program for people living with HIV in Thailand
Source: PLoS One. 2017 Sep 26;12(9):e0184986. doi: 10.1371/journal.pone.0184986 (PMC5614535; doi:10.1371/journal.pone.0184986)
Supplement: S1 File — contains a workshop agenda, sample of overview ICF-IPT project in Thailand, sample of IPT study, and sample of latent TB infection and TST. (PDF) Figure A. Workshop agenda. (PDF) Figure B. Sample of overview ICF-IPT project in Thailand. (PDF) Figure C. Sample of IPT study. (PDF) Figure D. Sample of latent TB infection and TST. (ZIP) [file pone.0184986.s002.zip › Figure A in S2 file. Workshop agenda.pdf]

AGENDA

Workshop on Isoniazid preventive therapy and Tuberculin skin test

****

08.30-09.00 am	Registration
09.00-09.30 am	Opening
	Overview of Intensified case finding (ICF)/ Isoniazid preventive therapy (IPT) project
09.30-10.30 am	Study in Chiang rai province
	-Evaluation of IPT among PLHIV in Chiang rai province
	-Is IPT among PLHIV essential in ART era?
10.30-10.45 am	Coffee break
10.45-12.00 am	Sharing lesson learned of TB screening and IPT in PLHIV
12.00-13.00 pm	Lunch
13.00-13.30 pm	Recording and reporting
13.30-14.30 pm	Latent TB infection and Tuberculin skin test (TST)
14.30-14.45 pm	Coffee break
14.45-15.45 pm	Hand on TST administration
15.45-16.30 pm	Q & A
	Summary and Closing
