# Supplementary material for: Implementing an isoniazid preventive therapy program for people living with HIV in Thailand
Source: PLoS One. 2017 Sep 26;12(9):e0184986. doi: 10.1371/journal.pone.0184986 (PMC5614535; doi:10.1371/journal.pone.0184986)
Supplement: S1 File — contains a workshop agenda, sample of overview ICF-IPT project in Thailand, sample of IPT study, and sample of latent TB infection and TST. (PDF) Figure A. Workshop agenda. (PDF) Figure B. Sample of overview ICF-IPT project in Thailand. (PDF) Figure C. Sample of IPT study. (PDF) Figure D. Sample of latent TB infection and TST. (ZIP) [file pone.0184986.s002.zip › Figure B in S2 file. Sample of overview ICF-IPT project in Thailand.pdf]

ICF/IPT project

(Project code 2/53-394)

23 July 2010

TB screening and diagnosis

“ICF/IPT”

- WHO, 2 of 3 I’s for TB/HIV
- ICF = Intensified case finding
- IPT = Isoniazid preventive therapy

Deviation of TB screening question

National

1. Cough greater than 2 weeks
2. Fever in the past 2 weeks
3. Weight loss >5%
4. Abnormal night sweats
5. Lymphadenopathy > 2cms

Proposed

1. Any cough in the previous 4 weeks
2. Any fever in the previous 4 weeks
3. Drenching night sweats lasting for 3 or more weeks in previous 4 weeks

Objective

General objective

- To evaluate the feasibility of using new algorithm for tuberculosis screening and diagnosis in people with HIV.

Specific objectives

- To evaluate the implementation of a new, evidence-based approach to TB screening and diagnosis in people with HIV
- To Assess clinician beliefs and perspectives about the utility and performance of the new algorithm

Participation

- 7 Participating sites
- 9 supporting TB laboratory

Methodology

PART 1

Evaluation of routine programmatic implementation of a TB screening and diagnostic algorithm

PART 2

Health care workers perceptions about using the TB screening and diagnostic algorithm

Today, skip part II

Part I

Phase I: July 01, 2010 – June30, 2011
Enrollment period
Phase II: July 01, 2011 – June 30, 2012
Follow up period

PART I
Study population

- Newly HIV infected patients who are presenting with lab report confirmation to a participating outpatient HIV care facility during July 01, 2010 to June30, 2011
- HIV patients aged at least 18 years old
- HIV patients have not yet initiated ART

Clinical Algorithm for Tuberculosis Screening in People with HIV

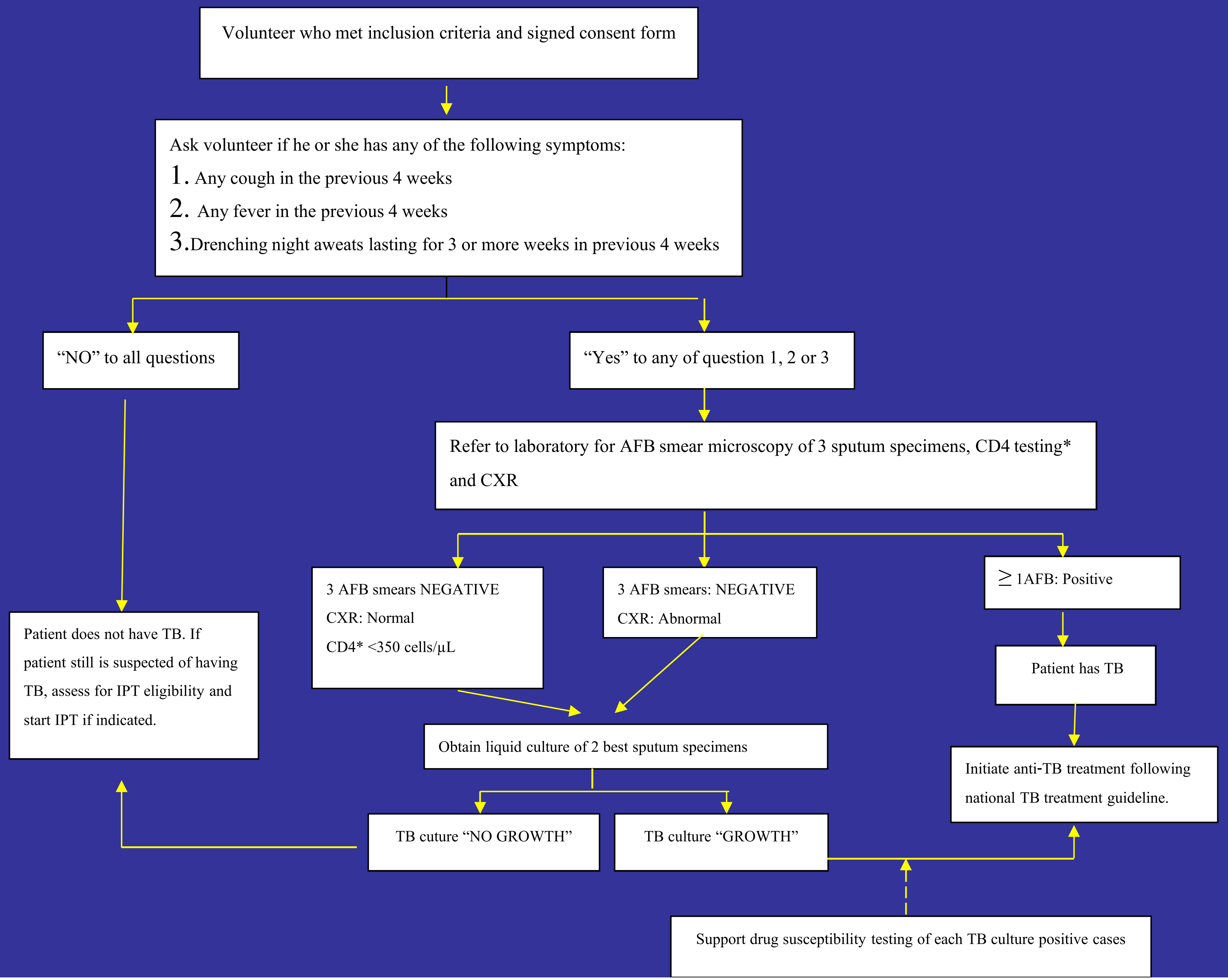

Diagnosis of Latent TB infection

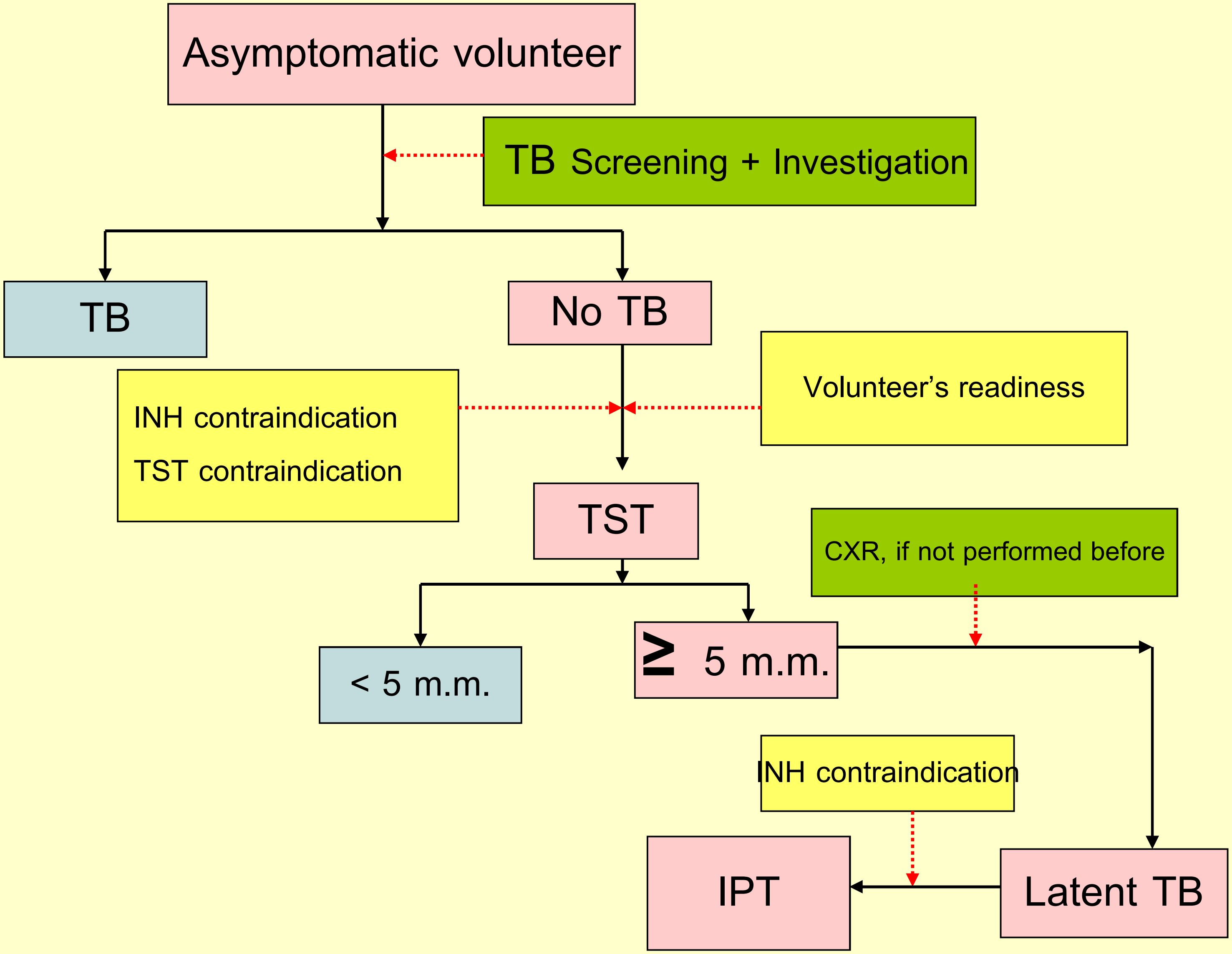

Timeline of all activities

**Activities**		**Time**
Stakeholder meeting		April 2010
Focus group for Clinicians		May 2010
SOP training		June 2010
Questionnaire for Clinicians		July 2010
Enrollment of PLWH		July 2010
Supervision		Every 3 months
Follow up meeting		January 2011
Follow up meeting<br>after 1-year enrollment		August 2011
Summary meeting:<br>Preliminary report<br>Lesson learned		August 2012

Acknowledgement

Taksin hospital
Sanpatong hospital
Maesod hospital
Srisaket hospital
MaharajNakhonSiThammarat hospital
Chiang Rai regional hospital
Bhudhachinaraj hospital
Somdejprajaotaksinmaharaj hospital
Vachira Phuket hospital

PHO: Chiang rai, Ubonratchathani, Tak, Phuket, Chiang Mai, Pitsanulok, Srisaket, Nakhon Si Thammarat

ODPC 7 Ubonratchathani
ODPC 10 Chiang mai
ODPC 9 Pitsanulok
ODPC 11 Nakhonsrithammarat

Research Institute of TB, Tokyo, Japan: RIT
Bangkok Metropolitan Administration (BMA)
Bureau of TB
Bureau of Aids TB and STI
Thailand MOPH-U.S. CDC collaboration (TUC)
