# Supplementary material for: Implementing an isoniazid preventive therapy program for people living with HIV in Thailand
Source: PLoS One. 2017 Sep 26;12(9):e0184986. doi: 10.1371/journal.pone.0184986 (PMC5614535; doi:10.1371/journal.pone.0184986)
Supplement: S1 File — contains a workshop agenda, sample of overview ICF-IPT project in Thailand, sample of IPT study, and sample of latent TB infection and TST. (PDF) Figure A. Workshop agenda. (PDF) Figure B. Sample of overview ICF-IPT project in Thailand. (PDF) Figure C. Sample of IPT study. (PDF) Figure D. Sample of latent TB infection and TST. (ZIP) [file pone.0184986.s002.zip › Figure C in S2 file. Sample of IPT study.pdf]

IPT study

-Evaluation of IPT among PLHIV in Chiang rai

-Is IPT among PLHIV essential in the ART era?

Jul 23, 2010

Proportion of Opportunistic infection in AIDS Cases in Thailand

Cases reported from 1984 – 2007

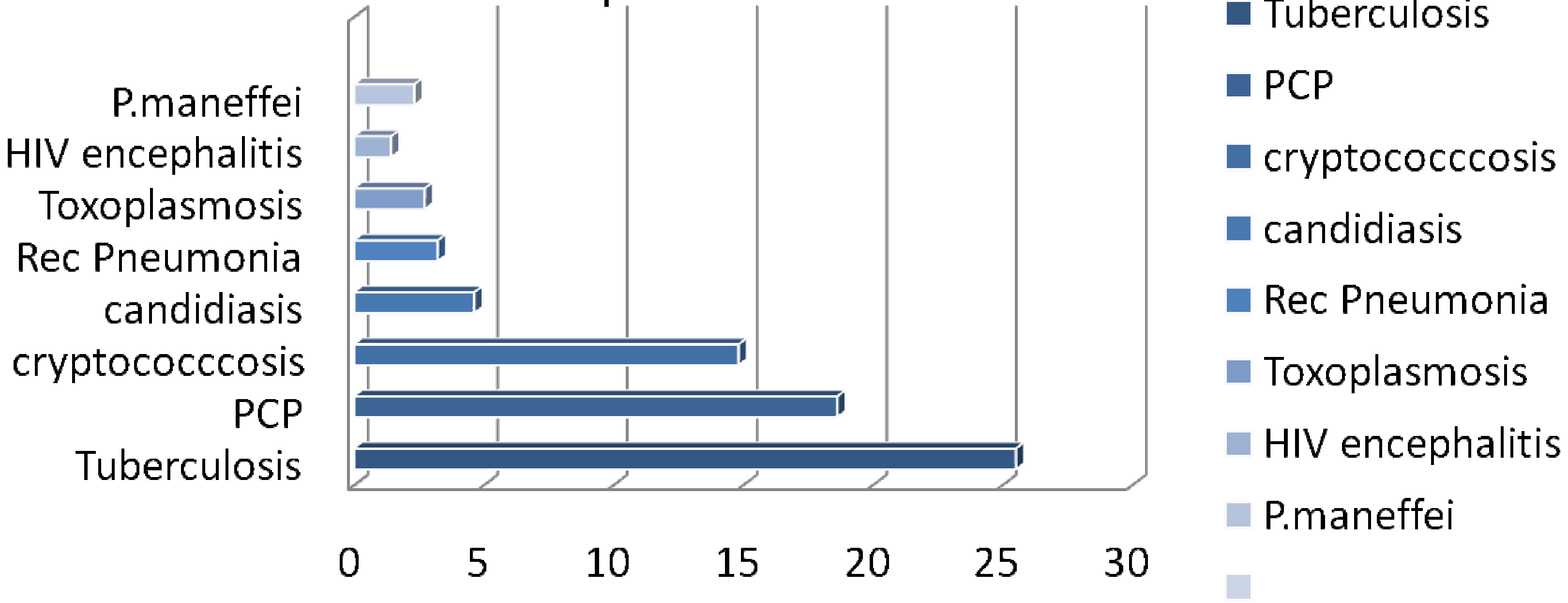

Source: Bureau of Epidemiology,MOPH,Thailand data as of February 28 , 2007

TB is the leading cause of death among PLHIV

HIV status	Lifetime risk of developing TB
Negative (-)	5-10%
Positive (+)	50%

Reference: TB/HIV: A clinical manual, WHO 2004

Risk group	case
1. AIDS case	170
2. Asymptomatic HIV infection	113

จาก **Self-Study Modules on TB, CDC, Atlanta** และ **Reichman L, Hershfield ES Tuberculosis: A Comprehensive International Approach** 1993)

Isoniazid preventive therapy (IPT)
has been known to reduce the risk of TB

IPT reduce the risk of active TB, 60-90%

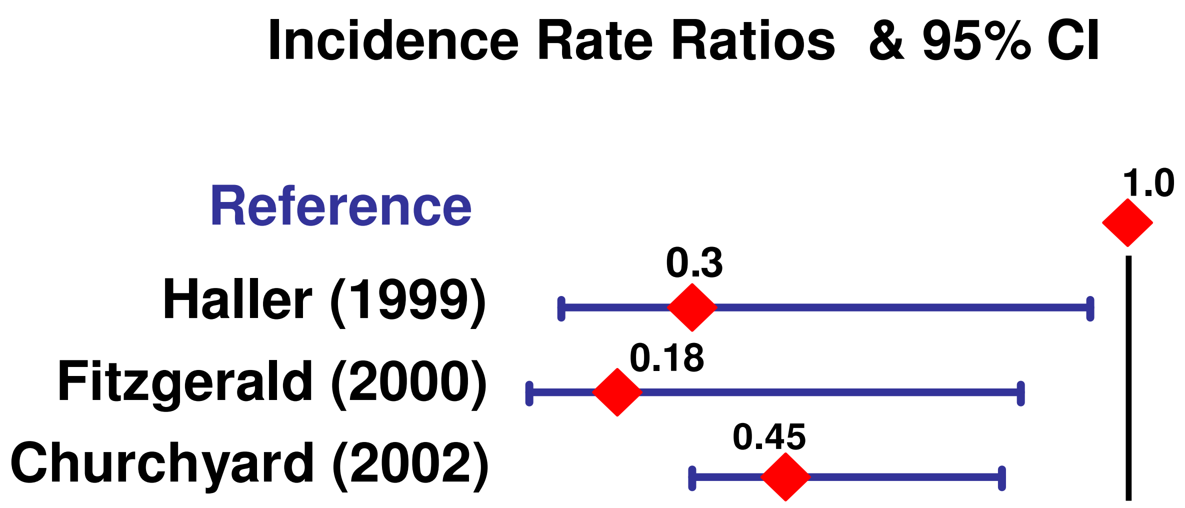

Woldehanna and Volmink, Cochrane Review 2006

Cochrane Review:

- 11 clinical trials, total PLHIV= 8,130 (randomized participants)
- **ในกลุ่มผู้ติดเชื้อทั้งหมด, INH ช่วยลดความเสี่ยงต่อการเกิดวัณโรค 36%**

  [Overall protective effect of RR 0.64 (95% CI 0.51, 0.81)]

- **ในกลุ่ม TST + , INH ช่วยลดความเสี่ยงต่อการเกิดวัณโรค 62%**

[Effect was greatest on those who were TST+ (RR 0.38; 95% CI 0.25, 0.57)]

- **ในกลุ่ม TST - INH ช่วยลดความเสี่ยงต่อการเกิดวัณโรค 17 %**

[Effect on those who were TST negative was not significant (RR 0.83; 95% CI 0.58, 1.18)]

SOURCE: Waldehanna S. Treatment of Latent Tuberculosis Infection in HIV Infected Persons (Review). *The Cochrane Collaboration*. Wiley Publishers. 2008

IPT prevent active TB

WHO/UNAIDS 1998 IPT Policy

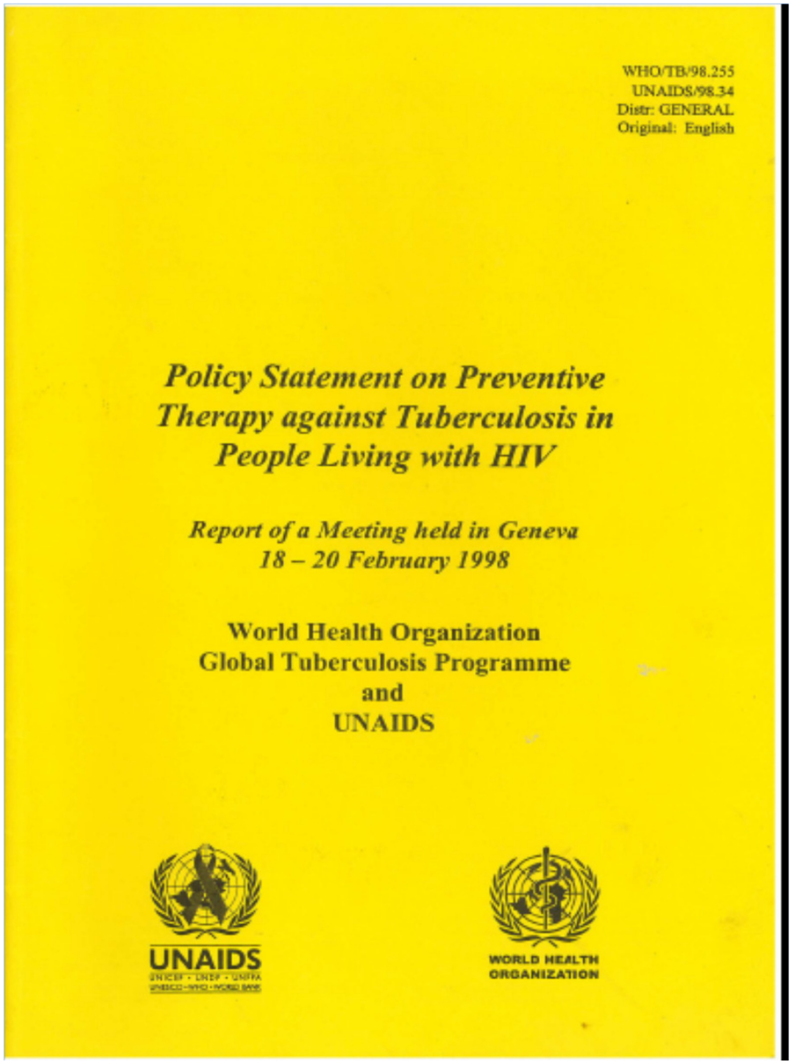

WHO/TB/98.255
UNAIDS/98.34
Distr: GENERAL
Original: English

Policy Statement on Preventive Therapy against Tuberculosis in People Living with HIV

Report of a Meeting held in Geneva
18 – 20 February 1998

World Health Organization
Global Tuberculosis Programme
and
UNAIDS

UNAIDS
UNICEF • UNDP • UNFPA
UNESCO • WHO • WORLD BANK

WORLD HEALTH ORGANIZATION

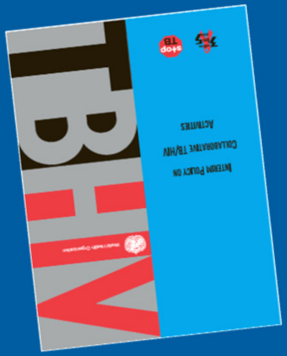

WHO 2004 policy on collaborative TB/HIV activities

A. Establish NTP-NACP collaborative mechanisms
- -Set up coordinating bodies for effective TB/HIV activities at all levels
- -Conduct surveillance of HIV prevalence among TB cases
- -Carry out joint TB/HIV planning
- -Monitor and evaluate collaborative TB/HIV activities

B. Decrease burden of TB among PLHIV (the "Three I's")
- -Establish intensified TB case finding
- **-Introduce INH preventive therapy**
- -Ensure TB infection control in health care and congregate settings

IPT
2547

C. Decrease burden of HIV among TB patients
- -Provide HIV testing and counselling
- -Introduce HIV prevention methods
- -Introduce co-trimoxazole preventive therapy
- -Ensure HIV/AIDS care and support
- -Introduce ARVs

Implementation progress

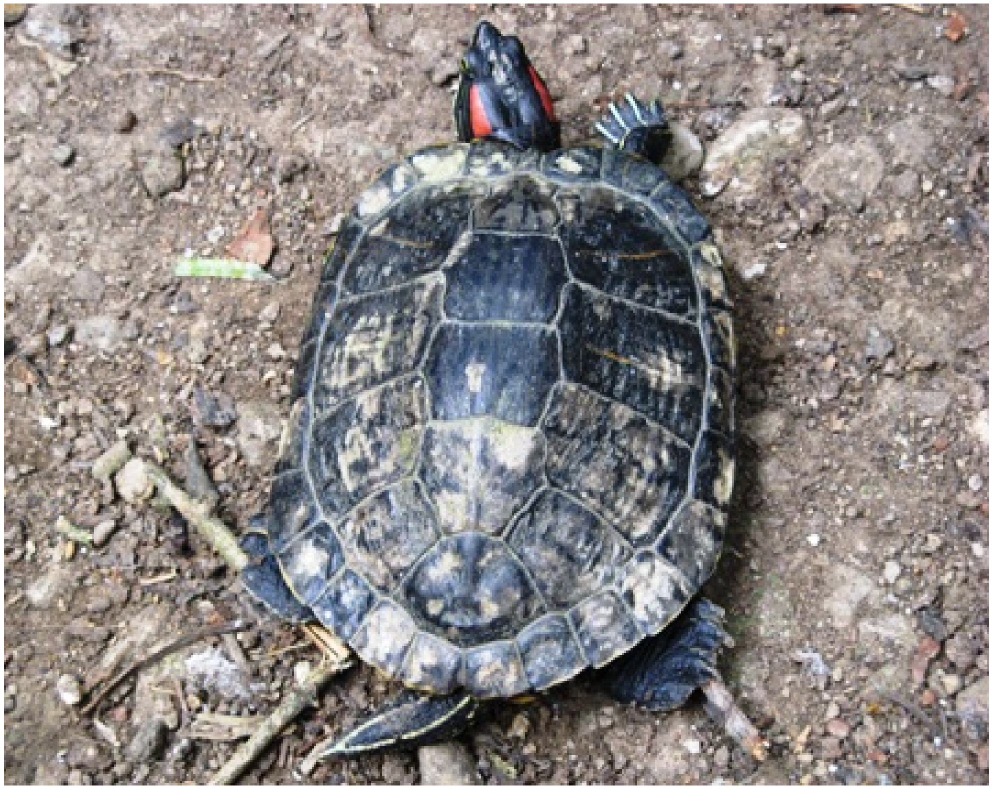

Issue concern of IPT

1. Screening for active TB
2. Drug resistance
3. Toxicity
4. Adherence
5. Duration of protection
6. Work load

ART reduce the risk of TB disease

Many studies show TB decreased in HIV infected person who started HAART.

South Africa:
ART associated with 64% reduction in TB incidence.

Brazil:
ART associated with 59% reduction in TB incidence.

Do we still need to implement IPT?

Does IPT have benefit for PLHIV in the ARV era?

ถึงแม้จะได้ยาต้านไวรัสแล้ว อุบัติการณ์การเกิดวัณโรคยังคงสูงอยู่ รวมทั้งในกลุ่มผู้ติดเชื้อเอชไอวี ที่ยังไม่ได้ยาต้านไวรัส

South Africa

HIV positive group	TB incidence rate Per 100 person year	95% CI
No IPT, No HAART	7.1	6.2 - 8.2
IPT	5.2	3.4 - 7.8
HAART	4.6	3.4 -6.2

Brazil

HAART	2.28	2.06-2.52
**20**-fold higher than general population of Brazil		

Thailand, Bamranaradura Institute

TB(48%) was major of opportunistic infection After initiation of ART.

TB incidence remains high during 1st year after ART initiation

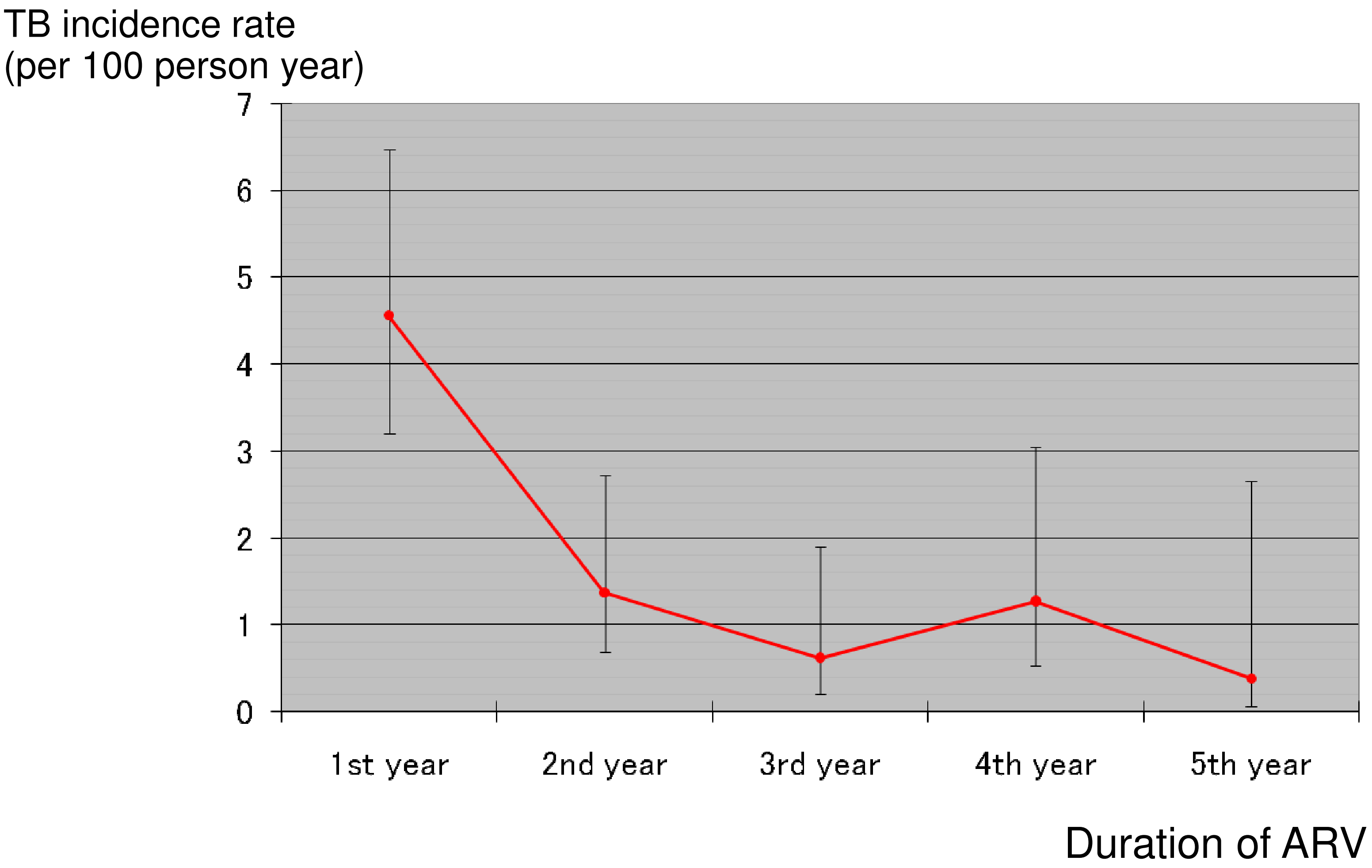

No information from South east Asia which have the second highest of number of people co-infection with TB and HIV.

Therefore, it is necessary to know whether the addition of IPT and HAART can more prevent TB in PLHIV or not?

Moreover, it is necessary to evaluate IPT program in Chiang Rai province.

Objective

1. To evaluate efficiency of IPT, HAART, IPT plus HAART in reduction of TB disease
2. To evaluate TB screening prior to IPT in Chiang rai province
3. To evaluate an IPT adherence in Chiang rai province
4. To evaluate the IPT toxicity in Chiang rai province
5. To evaluate the isoniazid resistance in Chiang rai province

Methodology

Prospective Cohort study of PLHIV:

- Study among PLHIV in 8 hospitals, Chiang rai
- Sep 2002- Aug 2009

IPT program

- PLHIV received IPT and recorded in IPT registration, 2003-2008

Preliminary result

TB episode during 2 year observation period of each phase

(preliminary results)

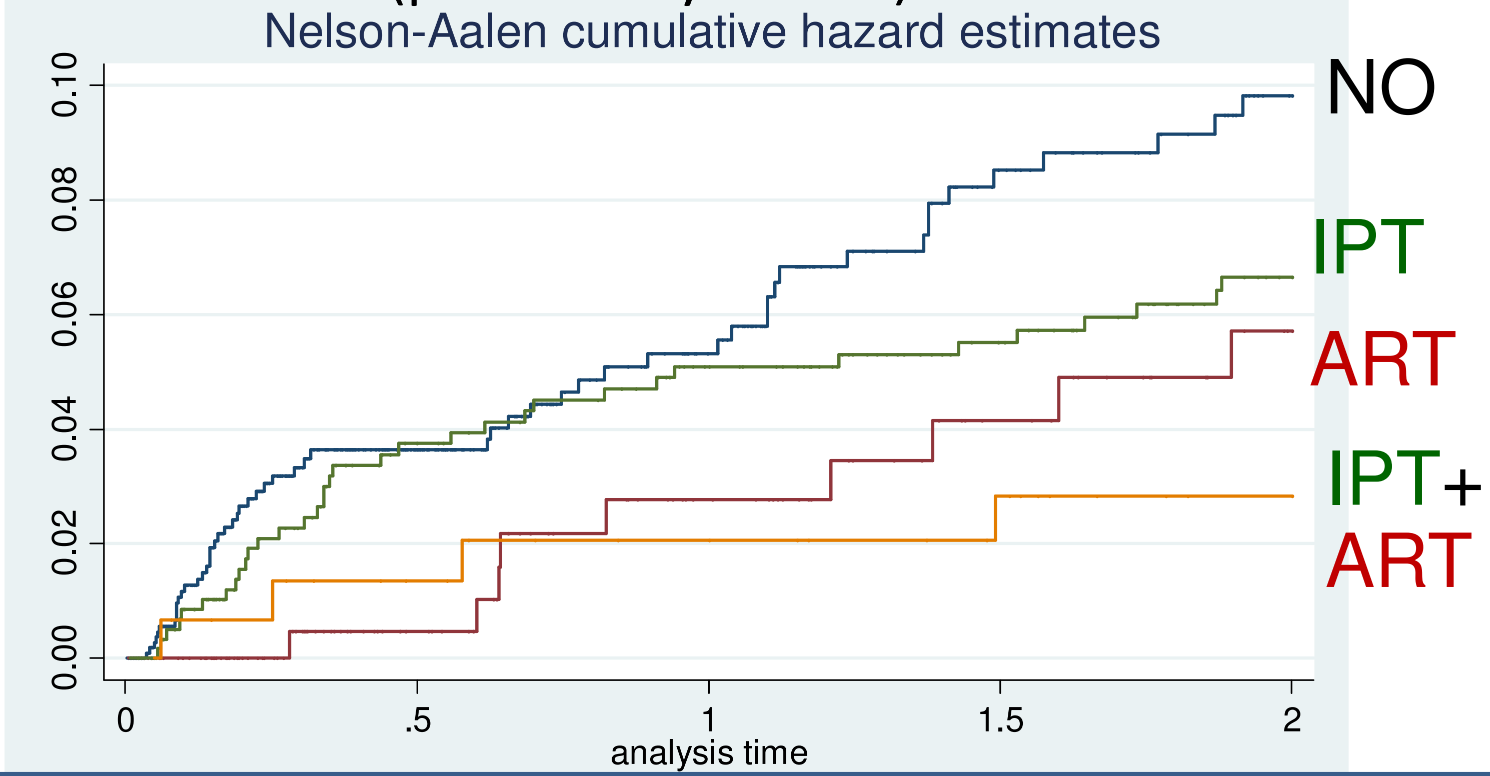

Phase	Period	Total	No.TB episodes	%	Person-years	Rate per 100py
0	No history of IPT and ART	1,185	56	4.7%	923.3	**5.8**
1	After starting IPT	258	9	3.5%	332.0	**2.7**
2	After starting ART	603	37	6.1%	999.5	**3.7**
3	After starting ART and IPT	150	4	2.7%	270.7	**1.5**

Rate ratio of TB episodes by Status of IPT and ART (preliminary results)

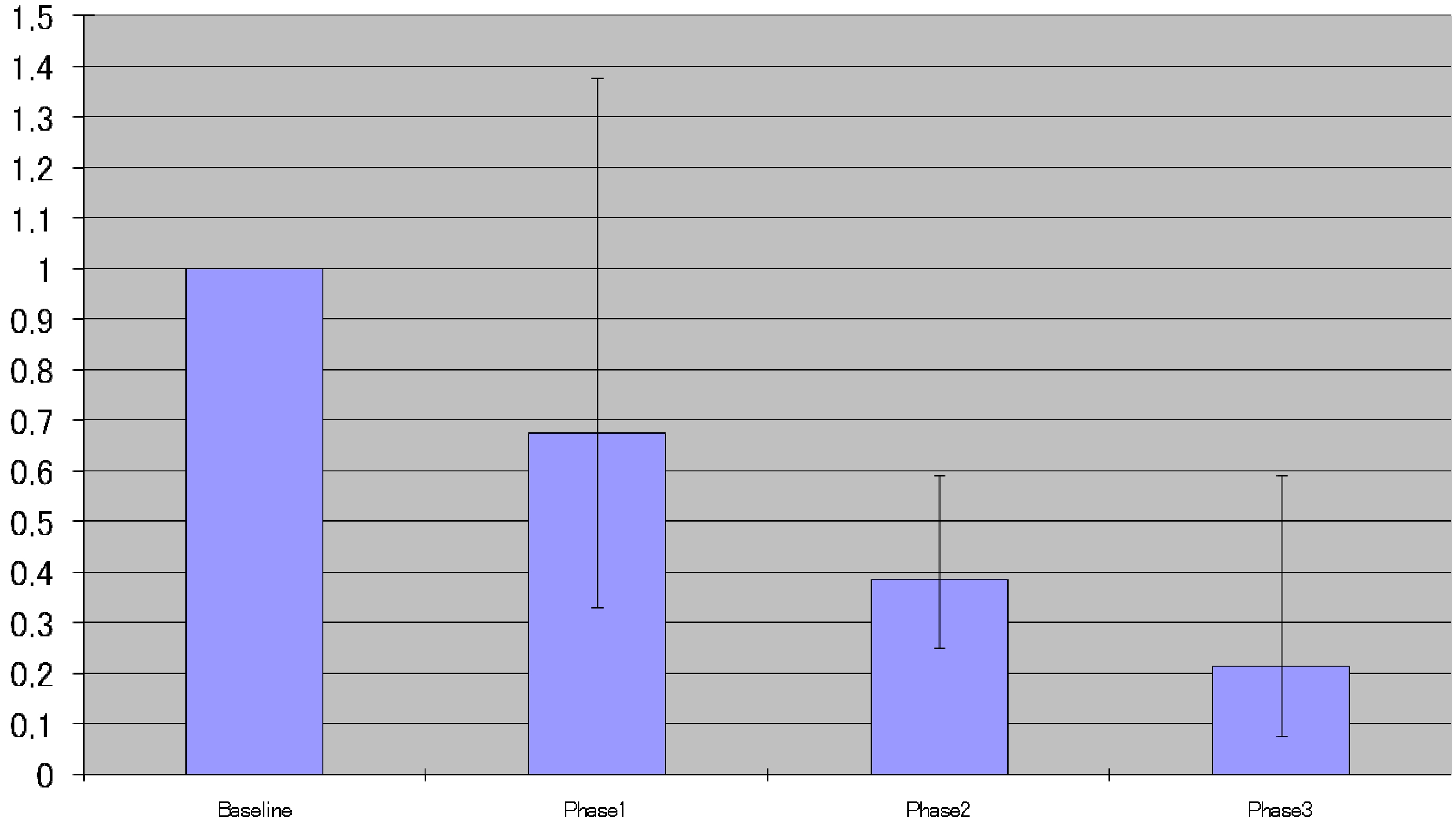

NO IPT ART IPT+ART

TB/HIV Research Project (RIT-JATA)

(adjusting for CD4 and Sex)

There are 2 studies in Brazil and south Africa indicate that TB was significantly reduced by IPT in HAART treated.

Isoniazid preventive therapy, HAART and tuberculosis risk in HIV-infected adults in South Africa: a prospective cohort

Jonathan E. Golub[a,b], Paul Pronyk[c,d], Lerato Mohapi[e], Nkeko Thsabangu[e], Mosa Moshabela[d], Helen Struthers[e], Glenda E. Gray[e], James A. McIntyre[e], Richard E. Chaisson[a,b] and Neil A. Martinson[a,e]

© 2009 Wolters Kluwer Health | Lippincott Williams & Wilkins

AIDS 2009, **23**:631–636

The impact of antiretroviral therapy and isoniazid preventive therapy on tuberculosis incidence in HIV-infected patients in Rio de Janeiro, Brazil

Jonathan E. Golub[a], Valeria Saraceni[b], Solange C. Cavalcante[b,c], Antonio G. Pacheco[b,c], Lawrence H. Moulton[a], Bonnie S. King[a], Anne Efron[a], Richard D. Moore[a], Richard E. Chaisson[a] and Betina Durovni[b,c]

© 2007 Lippincott Williams & Wilkins

AIDS 2007, **21**:1441–1448

TB incidence

	Brazil	South Africa	Chiang Rai	Brazil	South Africa	Chiang Rai
Exposure category	TB cases			Incidence Rate (per 100 PYs) (95% CI)		
Naïve	155	200	56	4.01 (3.40-4.68)	7.1 (6.2-8.2)	5.8 (4.45-7.51)
HAART only	221	44	9	1.90 (1.66-2.17)	4.6 (3.4-6.2)	2.7 (1.41-5.21)
Isoniazid only	5	22	37	1.27 (0.41-2.95)	5.2 (3.4-7.8)	3.7 (2.68-5.10)
Both	10	1	4	0.80 (0.38-1.47)	1.1 (0.2-7.6)	1.5 (0.55-3.92)
TOTAL	391	267	106	2.28 (2.06-2.52)		

TB rates by ARV and INH treatment status

Rio de Janeiro, Brazil, 2003-2005

Exposure category	Person-Years	TB cases	Incidence Rate (per 100 PYs) (95% CI)	Incidence Rate Ratio (95% CI)	Adjusted Hazard Ratio* (95% CI)
Naïve	3,865	155	4.01 (3.40-4.68)	REF	REF
HAART only	11,627	221	1.90 (1.66-2.17)	0.48 (0.39-0.59)	0.41 (0.31-0.54)
Isoniazid only	395	5	1.27 (0.41-2.95)	0.32 (0.10-0.76)	0.57 (0.18-1.82)
Both	1,253	10	0.80 (0.38-1.47)	0.20 (0.09-0.91)	0.24 (0.11-0.53)
TOTAL	17,140	391	2.28 (2.06-2.52)		

* Adjusted for age, sex, CD4, prior history of TB

Golub et al *AIDS* 2007, **21**:1441–1448

TB rates by ARV and INH treatment status South Africa, 2003-2005

Exposure category	Person-Years	TB cases	Incidence Rate (per 100 PYs) (95% CI)	Incidence Rate Ratio (95% CI)	Adjusted Hazard Ratio* (95% CI)
Naïve	2,815	200	7.1 (6.2-8.2)	REF	REF
HAART only	952	44	4.6 (3.4-6.2)	0.65 (0.46-0.91)	0.36 (0.25-0.51)
Isoniazid only	427	22	5.2 (3.4-7.8)	0.73 (0.44-1.13)	0.87 (0.55-1.36)
Both	93	1	1.1 (0.2-7.6)	0.15 (0.004-0.85)	0.11 (0.02-0.78)
TOTAL		267			

* Adjusted for age, sex, CD4, clinic location

Golub et al; AIDS 2009, Vol 23 No.5

INH resistance

-During 2002-2009, 2,274 PLHIV participated, 208 (10.9%) were found TB disease

-156 cases obtained sputum for TB culture, 11 cases were found INH resistance

9 cases, no IPT   2 cases, on IPT

IPT and Drug Resistant TB
(Balcell's 2006 meta-analysis)

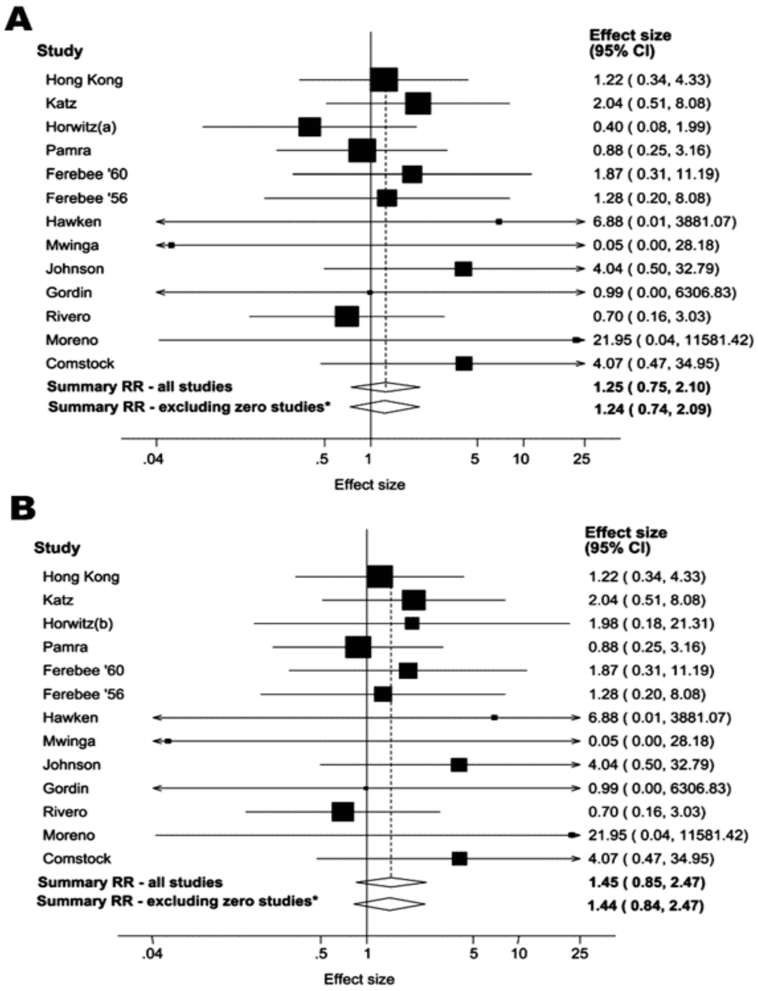

- **Review of 13 IPT trials**
- **~35,000 participants shows**
- **Concluded low risk of selecting resistance**

(RR 1.45, 95% CI 0.85-2.47)

no study that concluded IPT induce INH resistance

SOURCE: Balcells M. Isoniazid Preventive Therapy and Risk for Resistant Tuberculosis. *Emerging Infectious Diseases.* www.cdc.gov/eid. Vol 12, No. 5. May 2006

Difficulty of screening active TB

Year	Number	No. of case Develop TB	No. of month	Remark
2546 (2003)	**70**	**0**	-	
2547 (2004)	**157**	**4**	**0, 0, 5, 7**	CXR: reticulonodular infiltration (1)<br>No information (2)<br>TB LN at month 7
2548 (2005)	**105**	**4**	**1, 1, 3, 9**	cough 14 days, weight loss(1)<br>Fever, cough, weight loss(1)<br>Cough 4-5 days, weight loss (1)<br>No information (1)
2549 (2006)	**82**	**0**	-	
2550 (2007)	**187**	**1**	**0**	Had LN enlargement before
2551 (2008)	**151**	**0**	-	
**Total**	**752**	**9 (0.01%)**		

Toxicity

Year	Number	No. of case with drug toxicity	% drug toxicity	Remark
2546	70	0	0%	
2547	157	5	3%	Rash 1, N/V 2 , mild hepatitis , Uk 1
2548	105	1	1%	Oral ulcer
2549	82	1	1%	Oral ulcer
2550	187	6	3%	Rash 2, lab abnormal
2551	151	5	3%	Abnormal Lab, hepatitis rash 2, dizziness
Total	752	18	2%	

Isoniazid-related Hepatotoxicity

United States Public Health Service 1971-1973

13,838 persons on IPT

8 Deaths (7 in Baltimore; 0.0005%)

Centers for Disease Control 1972-1988

1,084,760 started on IPT

152 ***reported*** IPT-related deaths (0.00014%)

32 ***confirmed*** IPT-related deaths (0.00002%)

Death reports could not be verified due to lack of diagnostic markers for hepatitis

SOURCES: Snider D. Isoniazid-Associated Hepatitis Deaths: *A Review of Available Information. American Review of Respiratory Disease*. 1992; 145:494-497

Kopanoff D. Isoniazid-Related Hepatitis. A US Public Health Service Cooperative Surveillance Study. *American Review of Respiratory Disease*. Volume 117, 1978

Adherence

Year	Number	Complete (9 months)	default
2546	70	60 (86%)	5 (6%)
2547	157	130 (83%)	9 (8%)
2548	105	93 (89%)	8 (5%)
2549	82	74 (90%)	4 (3%)
2550	187	174 (93%)	5 (5%)
2551	151	138 (91%)	7 (1%)
Total	752	669 (89%)	38 (5%)

Treatment outcome of IPT program in Chiang Rai Province, 2003-2008

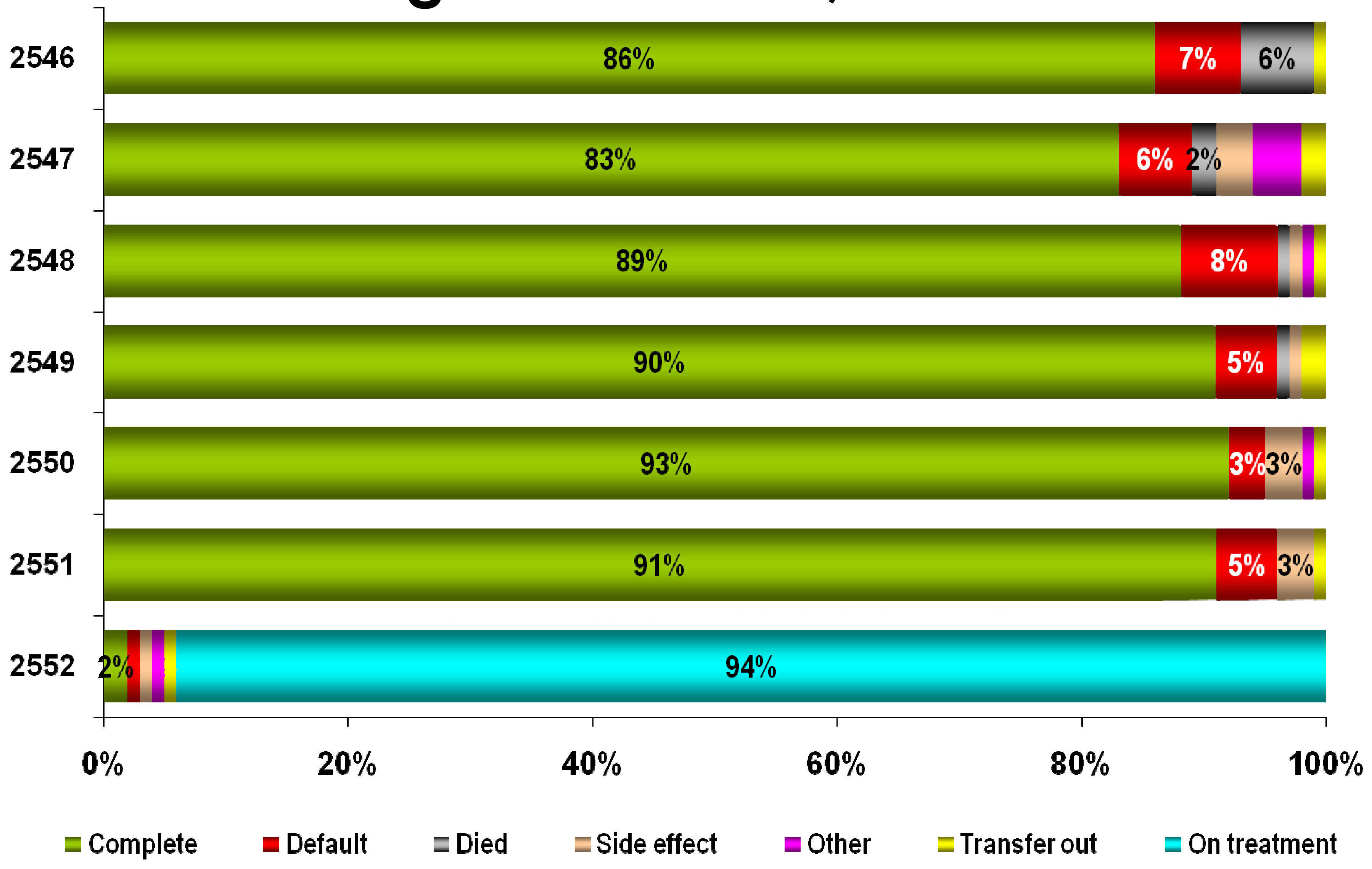

Thank you

Comment and suggestion
