# Supplementary material for: Implementing an isoniazid preventive therapy program for people living with HIV in Thailand
Source: PLoS One. 2017 Sep 26;12(9):e0184986. doi: 10.1371/journal.pone.0184986 (PMC5614535; doi:10.1371/journal.pone.0184986)
Supplement: S1 File — contains a workshop agenda, sample of overview ICF-IPT project in Thailand, sample of IPT study, and sample of latent TB infection and TST. (PDF) Figure A. Workshop agenda. (PDF) Figure B. Sample of overview ICF-IPT project in Thailand. (PDF) Figure C. Sample of IPT study. (PDF) Figure D. Sample of latent TB infection and TST. (ZIP) [file pone.0184986.s002.zip › Figure D in S2 file. Sample of latent TB infection and TST.pdf]

Mantoux Tuberculin Skin Test

23 July 2010

Tuberculin Skin Test

Testing for…..

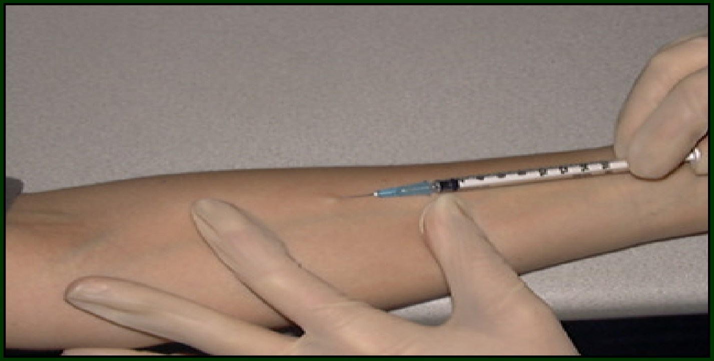

M. tuberculosis **Infection**

TB infection Vs TB disease

Topic	TB infection	TB disease
■ TB infection	Yes	Yes
■ TST result	Positive	Positive
■ CXR	Normal	(Mostly) Abnormal
■ Sputum microscopy	Negative	Positive
■ TB Symptom	No symptom	Had symptom
■ Transmission	Not transmit to other	may be transmit to other. Particularly, before TB treatment

copyright@TB/HIV Research Project (RIT-JATA)

Source: modified from Self-Study Modules on Tuberculosis, CDC, Atlanta, 1995, Module 1.p.12.

Tuberculin skin test (TST)

Type of response : delayed (cellular) hypersensitivity reaction of T-cell that had been activated from TB infection

Tuberculin

is reagent that has tuberculo-protein from cell wall of tubercle bacilli or *M. tuber-culosis*

Tuberculin

2 Types:

1. Koch’s old tuberculin (O.T.)
2. Purified protein derivatives (PPD) : PPD

Tuberculin PPD-TRC (Thai Red Cross)

Manufacturing by TRC

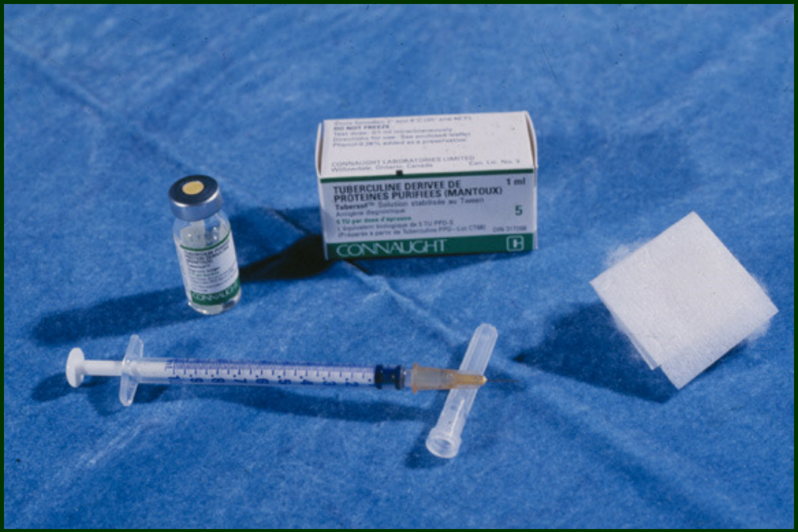

Dose 0.1 ml. - PPD 10 IU

Store at 2-8 °C

Protect from light

Don’t freeze

Tuberculin skin test

1. Multiple – puncture test

is technique that pass reagent into skin by using multiple – puncture device (MPD), 1 cm-plate with tine (see picture)

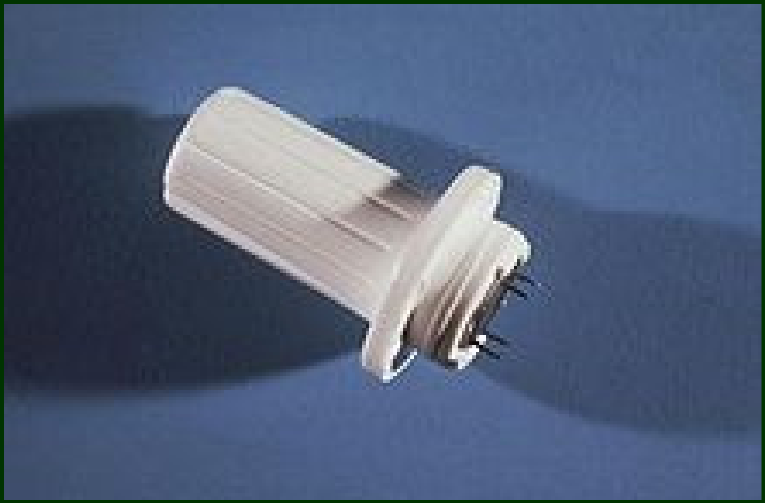

This method is less credible than Mantoux. It’s depend on press method, touch-skin duration, skin characteristic and moisture.

Tuberculin skin test

2. Mantuox test

In 1910, Mantoux recommended injecting reagent into arm. This technique is the most acceptable and continued using as standard practice

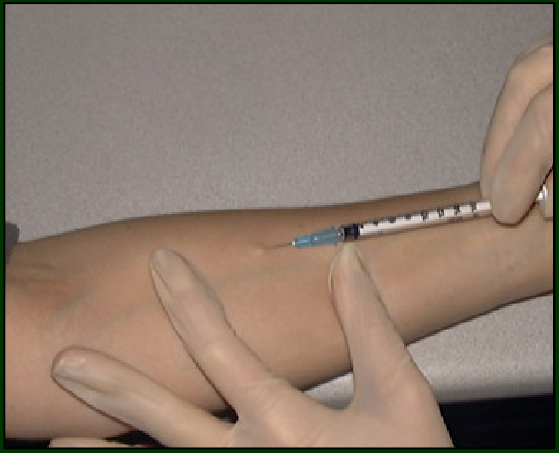

The Mantoux tuberculin skin test

Inject PPD reagent 0.1 ml into derm is, approximately 2-3 inches below arm brace, avoid scar or other lesions

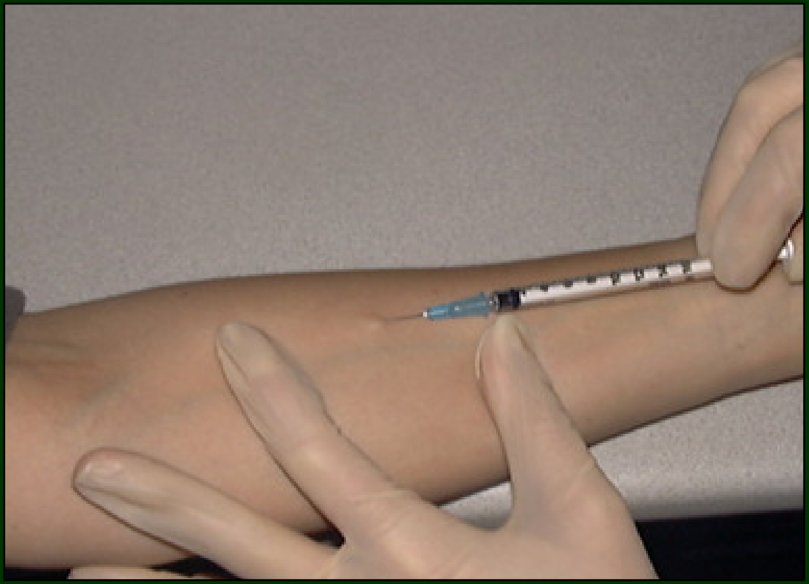

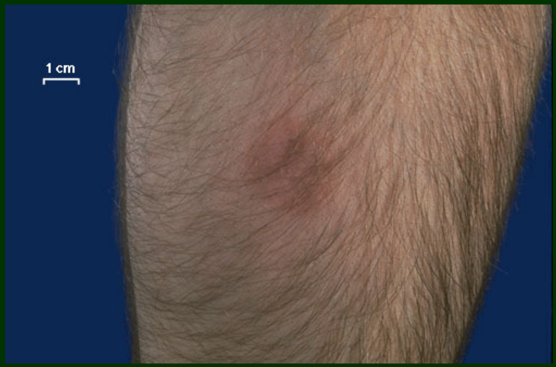

The Mantoux tuberculin skin test

Inject reagent into the dermis, just below the epidermis

Needle injection size: should be

25-29 G X½”

Syringe: Insulin syringe

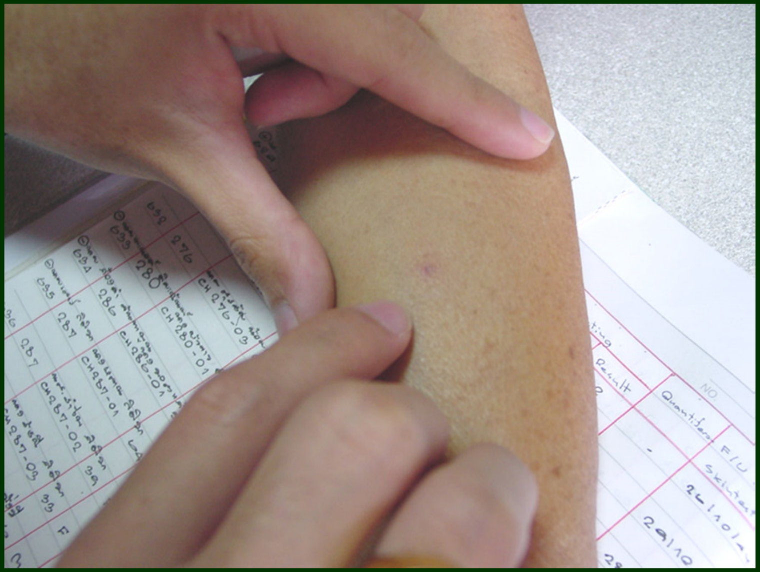

Tuberculin Skin Test(TST)

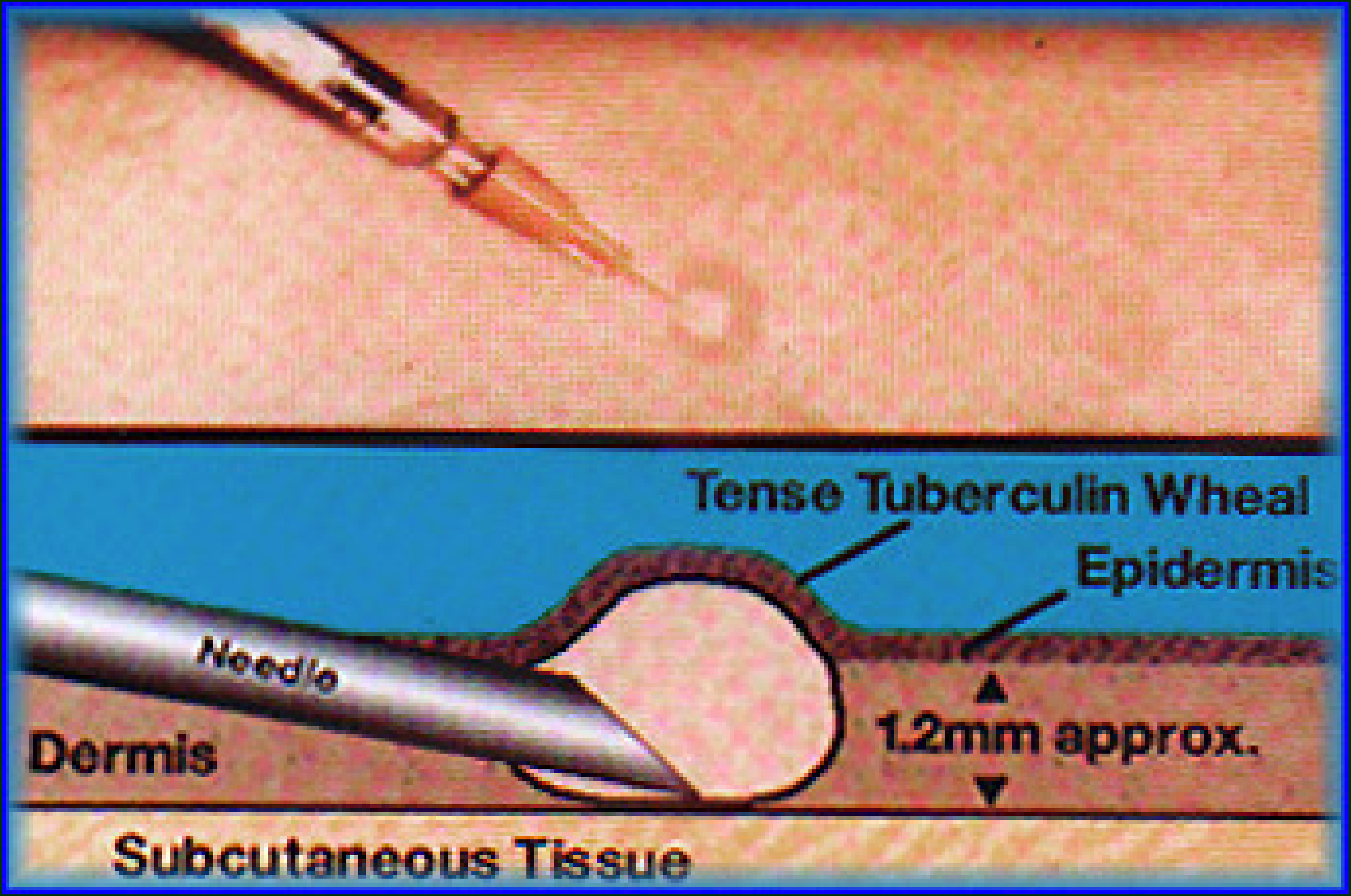

The Mantoux tuberculin skin test

Size 6-10 mm of blistering is required.

Re-inject on other arm, if has reagent leakage, or <6 mm of blistering during injection

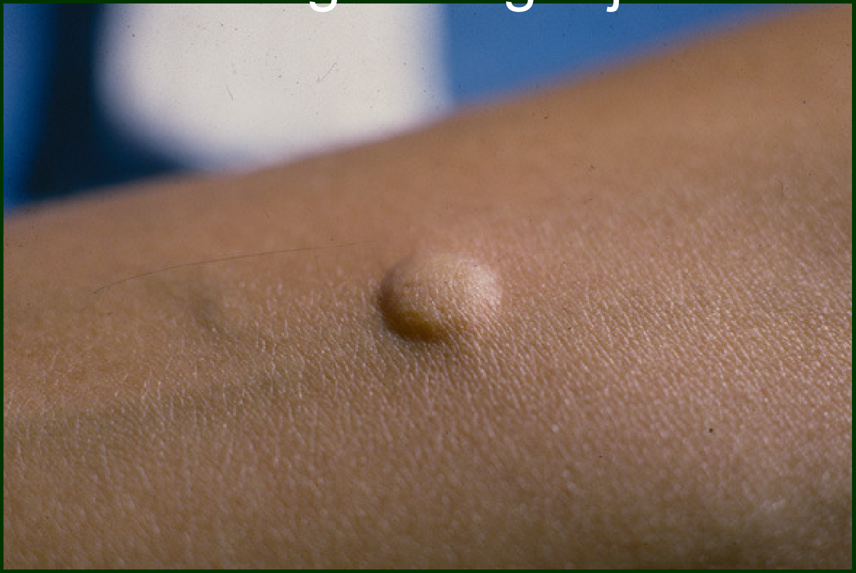

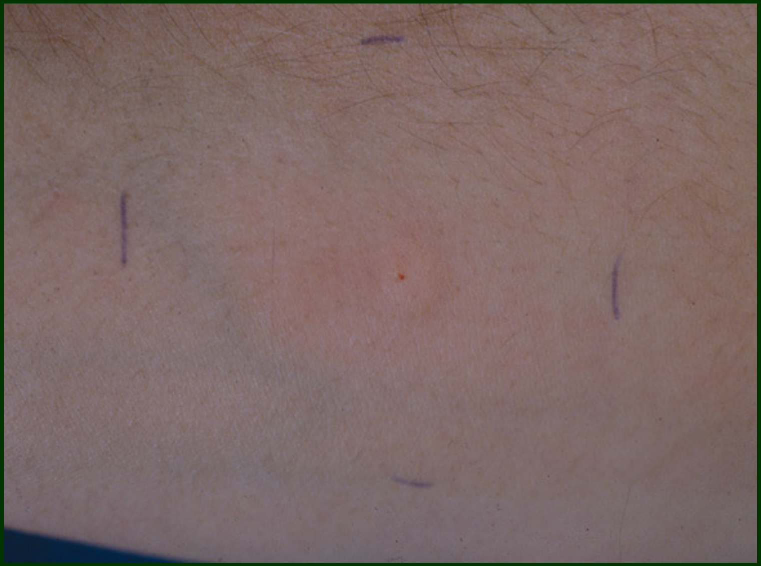

May be mark on around injecting site

Reaction on injecting site

- skin reaction will occur within 5-6 hours
- On injecting site, be found
  - Induration, greatest diameter between 48-72 hours
  - erythema
  - or vesicle

TST administer reading

- Read between 48-72 hours after administration
- Draw from outside to palpable, raised, harden area

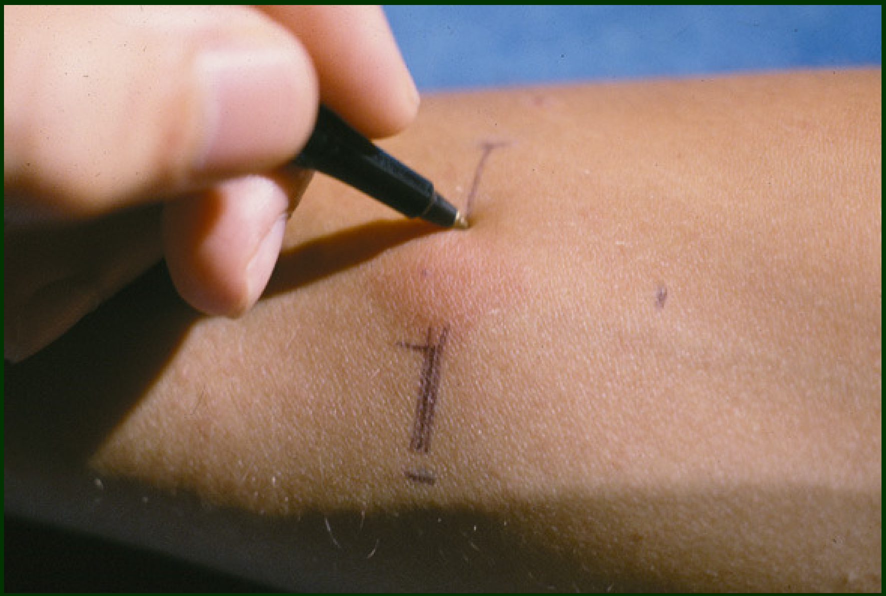

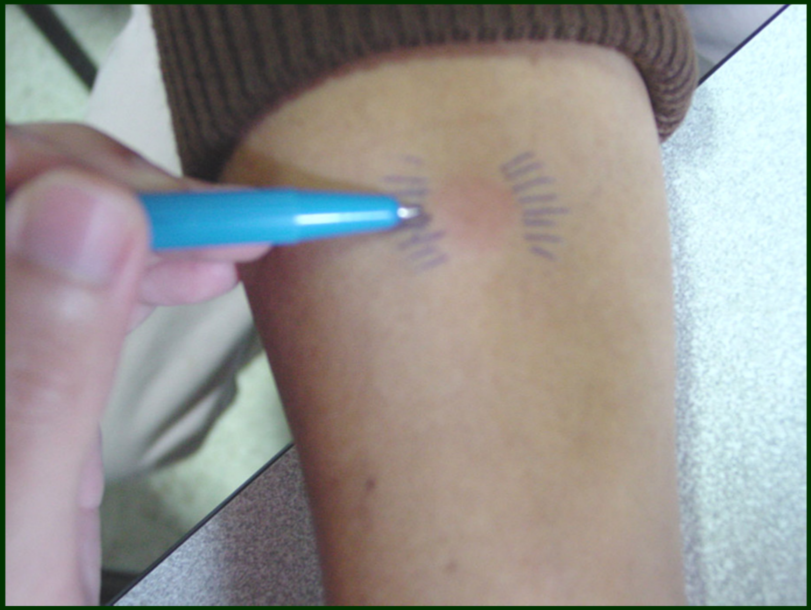

TST administer reading

- Diameter measurement

  - Record of measurement of induration in millimeter (mm)

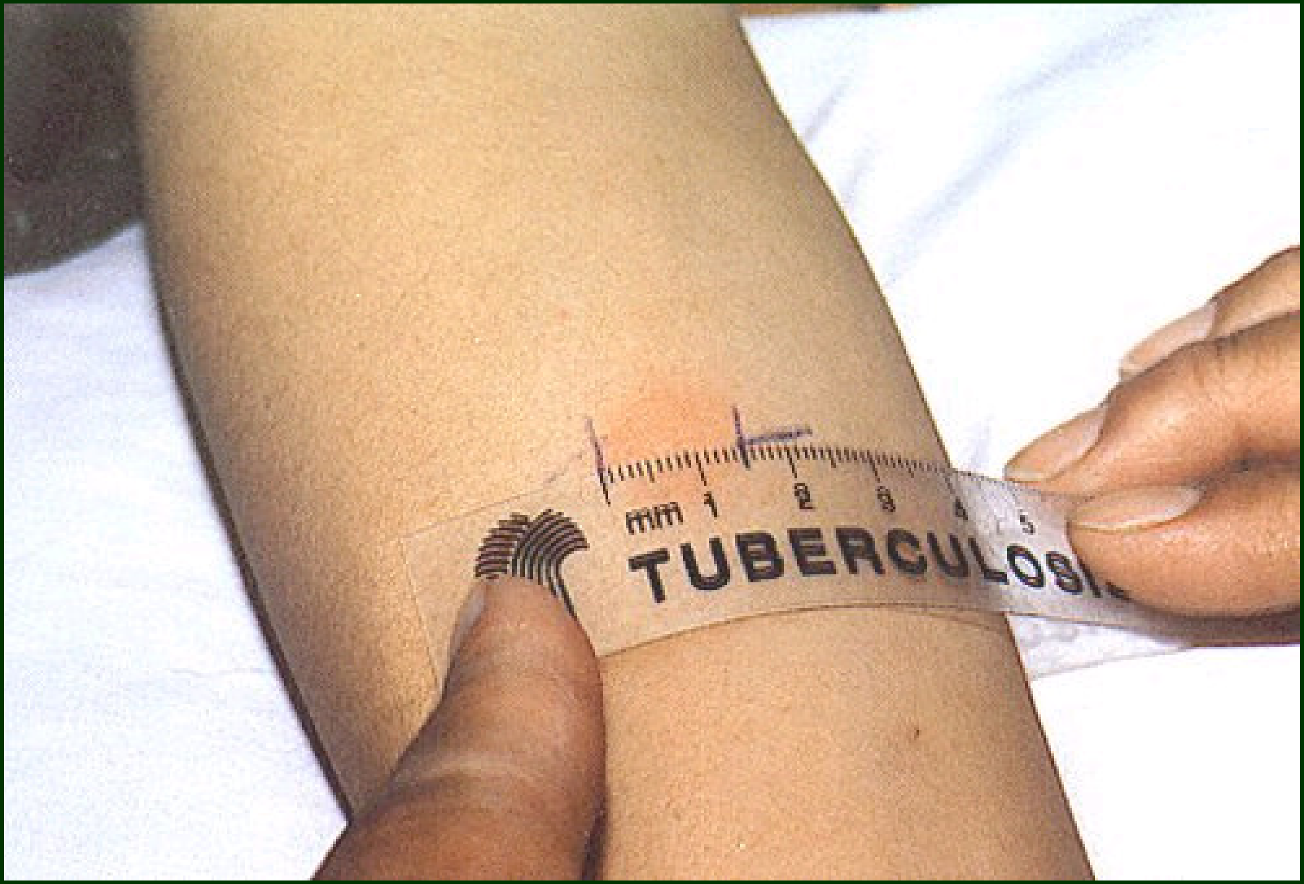

Measurement of TB infection

by using tuberculin skin test (TST)

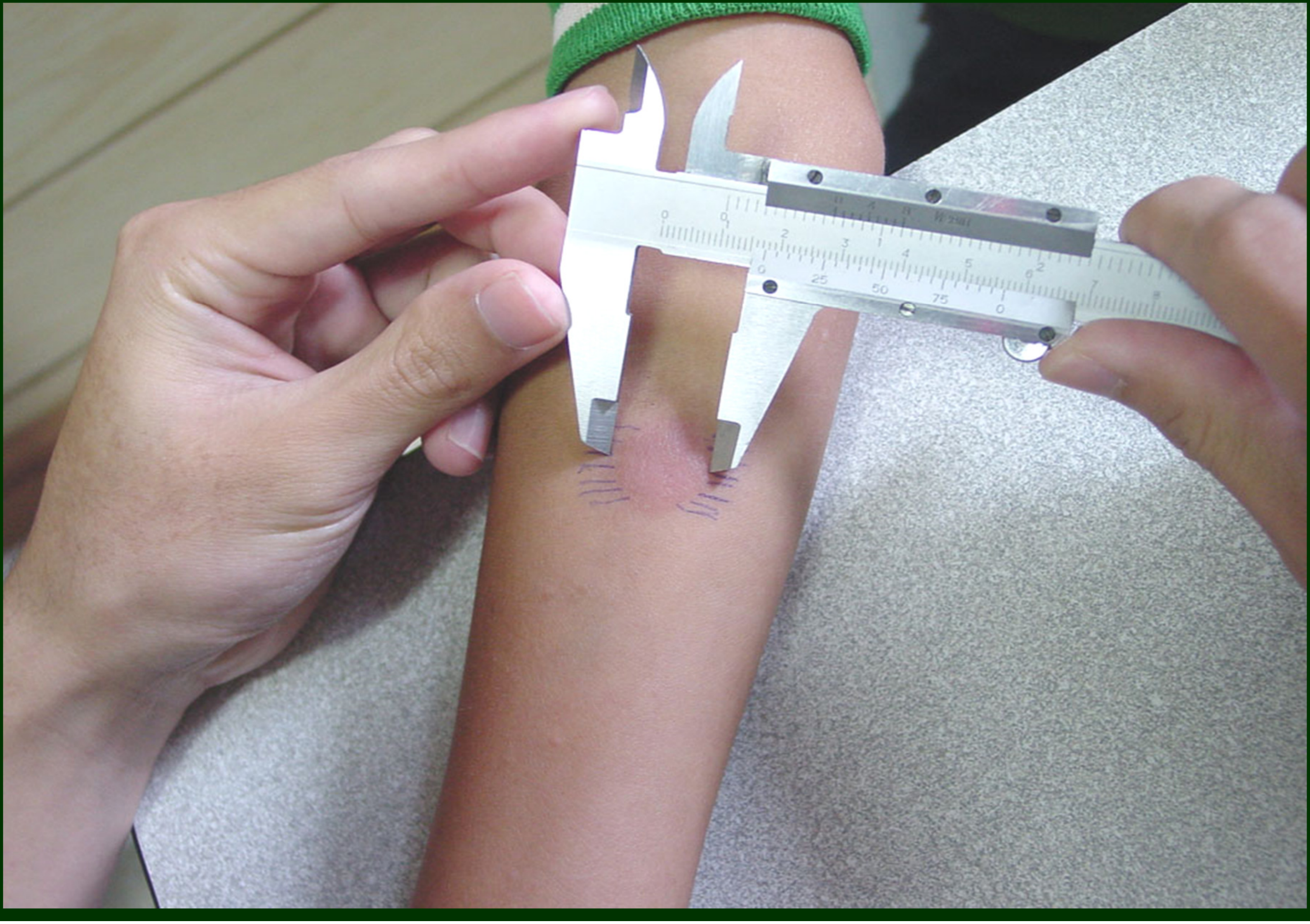

Reading TST

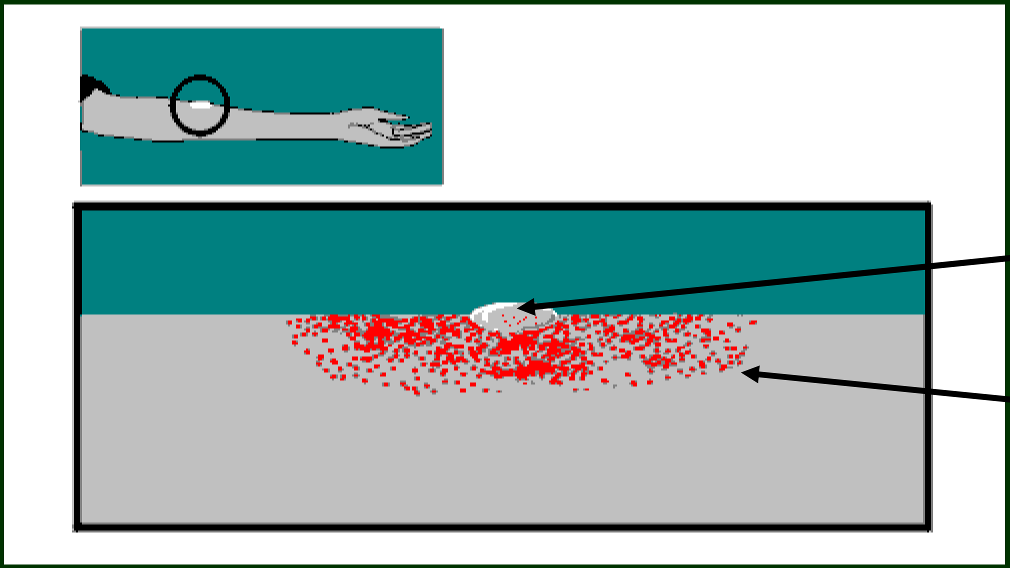

Induration (palpable, raised, harden or swelling area)

Erythema or redness

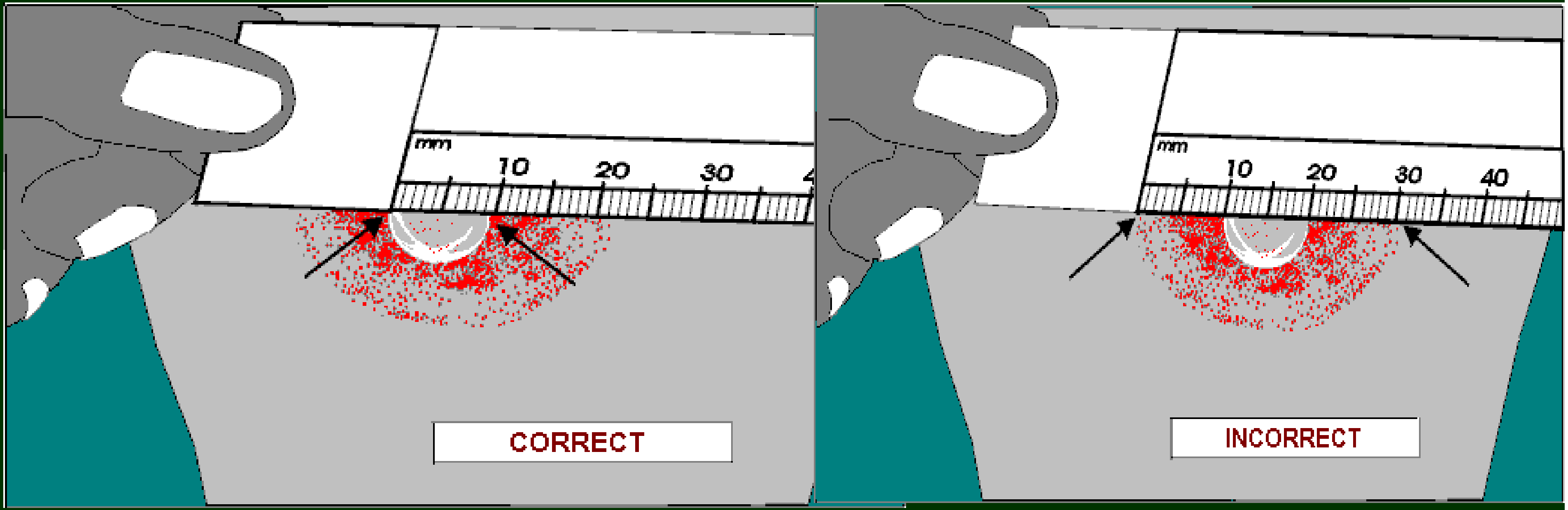

Reading the TST

- Positive TST reactions can be measured accurately for up to 7 days

Interpretation

Positive result

≥ 5 mm

- PLHIV
- TB Contact

≥ 10 mm

- Healthcare workers

≥ 15 mm

- General population

(Modified from Self-Study Modules on TB, CDC, Atlanta, Georgia, March 1995)

False positive result

False-Positive Reactions to the Tuberculin Skin Test

**Possible Cause**	**People at Risk**	**Action to Take***
Nontuberculous mycobacteria **(NTM)**	People infected with nontuberculous mycobacteria	Evaluate for TB disease if person has TB symptoms
**BCG** vaccination	People vaccinated with BCG	

(Modified from Self-Study Modules on TB, CDC, Atlanta, Georgia, March 1995)

False-Negative Reactions to the Tuberculin Skin Test

Possible Cause	People at Risk	Action to Take*
Anergy	HIV-infected people, other people with weakened immune systems	May do anergy testing
Recent TB infection	People infected with M. tuberculosis within the past 10 weeks	Retest 10 weeks after exposure to TB ended
Very young age	Children younger than 6 months old	Retest when child is 6 months old and 10 weeks after exposure to TB ended

(Modified from Self-Study Modules on TB, CDC, Atlanta, Georgia, March 1995)

False negative result

Factors of client

- Has viral, bacteria or fungal infection
- Recently get Lived-vaccination (e.g., MMR)
- Abnormal metabolism (e.g., chronic renal failure)
- Malnutrition (e.g., severe protein deficiency)
- Lymphoid system disease (e.g. lymphoma)
- Age, new born, elderly

False negative result

Factors associate to reagent

- inappropriate storage (e.g., expose to sunlight, heat)
- contaminated
- Chemical denaturation

Factors associate to administer

- less reagent amount
- subcutaneous injection, not intradermal injection
- delayed injection after draw from vial to syringe

False negative result

Factors associate to result reading and recording

- Lack of experience of TST reading
- Bias
- Inaccuracy recording

CD4 and TST result

CD4		TST < 5		TST ≥ 5	
0-100	(N=591)	456	(32%)	20	(9%)
101-200	(N=333)	247	(17%)	38	(17%)
201-300	(N=302)	215	(15%)	43	(19%)
301-400	(N=239)	182	(13%)	34	(15%)
401-500	(N=157)	120	(8%)	22	(10%)
≥ 501	(N=312)	216	(15%)	67	(30%)
Total	1934	1436	(100%)	224	(100%)
